# Carbohydrate Sequence of the Prostate Cancer-associated Antigen F77 Assigned by a Mucin *O*-Glycome Designer Array*[Fn FN2]

**DOI:** 10.1074/jbc.M114.558932

**Published:** 2014-04-21

**Authors:** Chao Gao, Yan Liu, Hongtao Zhang, Yibing Zhang, Michiko N. Fukuda, Angelina S. Palma, Radoslaw P. Kozak, Robert A. Childs, Motohiro Nonaka, Zhen Li, Don L. Siegel, Peter Hanfland, Donna M. Peehl, Wengang Chai, Mark I. Greene, Ten Feizi

**Affiliations:** From the ‡ Glycosciences Laboratory, Department of Medicine, Imperial College London, W12 0NN London, United Kingdom,; the § Department of Pathology and Laboratory Medicine, University of Pennsylvania, Philadelphia, Pennsylvania 19104-6082,; the ¶ Glycobiology Unit, Tumor Microenvironment Program, Sanford-Burnham Medical Research Institute, La Jolla, California 92037,; the ‖ Department of Chemistry, New University, 2829-516 Lisbon, Portugal,; ** Ludger Ltd., Culham Science Centre, Oxfordshire OX14 3EB, United Kingdom,; the ‡‡ Foundation of Haemotherapy Research, Institute of Experimental Haematology and Transfusion Medicine, University of Bonn, D-53127 Bonn, Germany, and; the §§ Department of Urology, Stanford University School of Medicine, Stanford, California 94305

**Keywords:** Antibody, Carbohydrate Structure, Glycolipid, Mass Spectrometry (MS), Prostate Cancer, O-Glycome Designer Array, Cancer-associated Antigen, Carbohydrate Microarray, Glycan Array, Neoglycolipid

## Abstract

Monoclonal antibody F77 was previously raised against human prostate cancer cells and has been shown to recognize a carbohydrate antigen, but the carbohydrate sequence of the antigen was elusive. Here, we make multifaceted approaches to characterize F77 antigen, including binding analyses with the glycolipid extract of the prostate cancer cell line PC3, microarrays with sequence-defined glycan probes, and designer arrays from the *O*-glycome of an antigen-positive mucin, in conjunction with mass spectrometry. Our results reveal F77 antigen to be expressed on blood group H on a 6-linked branch of a poly-*N*-acetyllactosamine backbone. We show that mAb F77 can also bind to blood group A and B analogs but with lower intensities. We propose that the close association of F77 antigen with prostate cancers is a consequence of increased blood group H expression together with up-regulated branching enzymes. This is in contrast to other epithelial cancers that have up-regulated branching enzymes but diminished expression of H antigen. With knowledge of the structure and prevalence of F77 antigen in prostate cancer, the way is open to explore rationally its application as a biomarker to detect F77-positive circulating prostate cancer-derived glycoproteins and tumor cells.

## Teaser

**Background:** F77 is a previously uncharacterized prostate cancer-associated antigen.

**Results:** Using a microarray of sequence-defined glycolipids and neoglycolipids, mucin-*O*-glycan designer arrays, and mass spectrometry, we demonstrate that F77 antibody recognizes blood group H on 6-linked branches of poly-*N*-acetyllactosamine backbones.

**Conclusion:** F77 antigen is expressed on glycolipids and on glycoprotein *O*-glycans.

**Significance:** F77 antigen can now be explored rationally as a cancer biomarker.

## Introduction

Cancer-specific cell surface antigens are highly sought for diagnosis, imaging, and targeted therapy. Among such long sought biomarkers are altered carbohydrates of cell surface glycoproteins and glycolipids on malignant cells (1–4). The murine mAb F77, IgG3 subtype, was raised against the prostate cancer cell line PC3 (5, 6) and found to recognize both androgen-independent (PC3, PC3-MM2, and DU 145) and androgen-dependent (LNCaP) human prostate cancer cells, with little or no binding to nonprostatic cells. The mAb F77 was found to label >90% of primary prostate cancers in immunohistochemical studies, with only limited focal staining in benign prostate tissues, and 29 of 34 prostate cancer metastases were F77-positive.

Transformation of a benign prostatic cell line, RWPE-1, containing a small subpopulation (<10%) of F77-positive cells, with the constitutively active *K-ras* oncogene, created a tumorigenic F77-positive cell line, RWPE-2 (6). Furthermore, although F77-negative RWPE-1 cells had limited tumorigenic potential, F77-positive RWPE-1 cells were as tumorigenic as RWPE-2 cells, confirming an association between F77 antigen expression and malignancy. Treatment of mice bearing established prostate cancer xenografts with mAb F77 significantly inhibited growth, with no activity against an F77-negative xenograft (6). Altogether, these findings suggested that the F77 antigen is a novel, biologically important, and clinically relevant prostate cancer-associated molecule.

Efforts to identify the nature of the antigenic determinant of mAb F77 by immunoprecipitation of PC3 cell extracts with mAb F77 followed by gradient SDS-PAGE revealed a carbohydrate-rich component (<5 kDa) that was not stained by Coomassie Blue (6). A dose-dependent decrease of F77 antigen expression was observed in PC3 and DU 145 cells after treatment with the glycolipid synthase inhibitor 1-phenyl-2-palmitoylamino-3-morpholino-1-propanol but not with the protein *N*- and *O-*glycosylation inhibitors tunicamycin and benzyl-α-GalNAc, respectively. ELISA-based analyses indicated that lipid extracts of PC3 cells contained components with F77 antigen activity. Collectively, these data indicated that glycolipids are the main carriers of F77 antigen in PC3 cells, but the identity of the F77 antigen was not resolved.

Here, we describe a multifaceted approach to biochemical characterization of the F77 antigen. We have corroborated the expression of the antigen on glycolipids by microarray screening analyses with sequence-defined, lipid-linked glycan probes and by antigenic analysis of the PC3 lipid extract resolved by high performance thin layer chromatography (HPTLC).^7^ We found that the F77 antigen is also expressed on certain epithelial mucins, particularly strongly on porcine stomach mucin (PSM). This opened the way to the “designer” microarray approach (7), the term used for arrays of neoglycolipids (NGLs) (8) derived from ligand-bearing glycomes to reveal the oligosaccharide ligands they harbor, so that these can be isolated and characterized. We isolated from PSM an F77 antigen-positive *O*-glycan branch, determined the sequence by mass spectrometry (MS), and validated it by using a new custom microarray of sequence-defined glycolipids. In the accompanying article (57) a complementary approach of glycosyltransferase transfections has been used to characterize the F77 antigen.

## EXPERIMENTAL PROCEDURES

### 

#### 

##### Glycoproteins

Thirty preparations of mucin-rich glycoproteins were examined (Table 1). These included samples derived from ovarian cystadenoma fluids from collections of Winifred Watkins and Walter Morgan (samples 1–17 and 23) and Elvin A. Kabat (samples 24–27); meconia (samples 18–22), PSM, bovine submaxillary mucin purchased from Sigma, and a human cancer-associated glycoprotein designated HCA (gift of Dr. Joe Zhou, Egenix).

**TABLE 1:** **Mucin-type epithelial glycoproteins arrayed**

Types of glycoproteins and positions	Designation	Donor red cell blood group	Secretor status*^a^*
**Enriched cystadenoma glycoproteins***^b^*			
1	Cys-350 (Trp)*^c^*	A1	Nonsecretor
2	Cys-444 (Trp)	Unknown	Nonsecretor
3	Cys-444 (Pro)	Unknown	Nonsecretor
4	Cys-446 (Pro)	B	Nonsecretor
5	Cys-461 (Trp)	Unknown	Nonsecretor
6	Cys-461 (Pro)	Unknown	Nonsecretor
7	Cys-654 (Pro)	B	Nonsecretor
8	Cys-705 (Pro)	Unknown	Nonsecretor
9	Cys-717 (Pro)	A1	Nonsecretor
10	Cys-733 (Pro)	A1	Secretor
11	Cys-745 (Pro)	A	Nonsecretor
12	Cys-754 (Pro)	Unknown	Nonsecretor
13	Cys-756 (Trp)	A	Nonsecretor
14	Cys-756 (Pro)	A	Nonsecretor
15	Cys-762 (Trp)	O	Nonsecretor
16	Cys-762 (Pro)	O	Nonsecretor
17	Cys-765 (Pro)	O	Nonsecretor

**Meconia*^d^***			
18	Meconium B (Pro)	B	Unknown
19	Meconium Mo (Pro)	Unknown	Unknown
20	Meconium Wo (Pro)	Unknown	Unknown
21	Meconium He (Pro)	Unknown	Unknown
22	Meconium Pa (Pro)	Unknown	Unknown

**Purified cystadenoma glycoproteins**			
23*^b^*	B substance (Pro)	B	Secretor
23*^e^*	Og 10% 2× (Pep)	Unknown	Nonsecretor
25*^e^*	Og 10% from 20% (Pep)	Unknown	Nonsecretor
26*^e^*	N1 20% (Pep)	Unknown	Nonsecretor
27*^e^*	Tij 10% 2× (Pep)	B	Secretor

**Other glycoproteins**			
28	HCA	Unknown	Unknown
29	Porcine gastric mucin	A + H	Sigma
30	Bovine submaxillary mucin	Unknown	Sigma

*^a^* Nonsecretor refers to the lack of blood group A, B, or H antigens in donor saliva or cystadenoma fluid (recorded in archives).

*^b^* Samples 1–17 and 23 were lyophilized ovarian cystadenoma fluids from the collection of Winifred Watkins and Walter Morgan at the former Lister Institute. They were enriched for mucin-type glycoproteins by treatment at 37 °C for up to 16 h with trypsin (Sigma, T1426, l-1-tosylamide-2-phenylethyl chloromethyl ketone-treated) or Pronase (Roche Applied Science, 10165921001). After the reaction, the samples were centrifuged (4000 × *g* for 10–20 min); the supernatants were lyophilized, taken up in 3.5 mg/ml sodium acetate, and precipitated with ethanol, 80% (v/v). Sample 23 had been further purified by phenol extractions (53).

*^c^* Trp, Pro, and Pep refer to trypsin, Pronase, or pepsin enzyme treatments for solubilizing mucin-type glycoproteins.

*^d^* Samples 18–22 were from meconium and enriched after Pronase digestion and ethanol precipitation (54).

*^e^* Samples 24–27 were purified ovarian cystadenoma glycoproteins from the Elvin A. Kabat collection (Columbia Medical Center, New York). These had been pepsin-treated and precipitated with various concentrations of ethanol (55, 56).

##### Oligosaccharides

The carbohydrate sequences of these oligosaccharides are described under “Results.” The following oligosaccharides were from Elicityl (Crolles, France): lacto-*N*-neofucopentaose I (LnNFP I), the type 1-based blood group A hexaose (A-hexa-T1) and blood group B hexaose (B-hexa-T1); the type 2-based blood group H tetraose (H-tetra-T2), blood group A pentaose and hexaose (A-penta-T2 and A-hexa-T2, respectively), and blood group B pentaose and hexaose (B-penta-T2 and B-hexa-T2, respectively); and Lewis X tetraose (LeX-tetra) and Lewis Y pentaose (LeY-penta). These were purified as necessary by normal phase HPLC using an XBridge amide column (Waters) with a solvent system of acetonitrile/H_2_O (solvent A: acetonitrile/H_2_O, 80:20; solvent B: acetonitrile/H_2_O, 20:80) by gradient elution (25% solvent B to 33% solvent B in 33 min) with a flow rate of 0.7 ml/min and detection by UV light at 196 nm. The following oligosaccharides were from Dextra (Reading, UK): lacto-*N*-fucopentaose I (LNFP I), monofucosyllacto-*N*-hexaose I (MFLNH-I), monosialyl difucosyllacto-*N*-neohexaose, trifucosyl(1–2,1–2,1–3)-*iso*-lacto-*N*-octaose [TFiLNO (1–2,2,3)]. The oligosaccharides referred to as Orsay-1 (trisaccharide) and Orsay-6 and Orsay-7 (pentasaccharides) were chemically synthesized in the laboratory of the late Professor S. David (9). The purities of oligosaccharides were corroborated by MS.

##### Glycolipids and Nonfluorescent NGLs

The carbohydrate sequences of these are described under “Results.” A glycolipid extract of prostate cancer cell line PC3 (5 × 10^8^ cells) was prepared as described (6). Glycosylceramides crucial in the assignment of F77 specificity are shown in Table 2 and include the following: blood group A-active glycolipids octa- and tetradecaglycosylceramide (designated Ab and Ad, respectively) (10); blood group H-active glycolipids hepta- and decaglycosylceramide (designated H_2_ and H_3_, respectively) (11); blood group B-active glycolipids dodeca- and tetradecaglycosylceramide (designated BIII and BIV, respectively) (12); B-like decaglycosylceramide (13); and I-active octaglycosylceramide (14, 15). Impurities in glycolipid fractions containing Ab, Ad, and H_2_ were removed by semi-preparative HPTLC. Nonfluorescent NGLs for a new focused microarray (designated F77/Ii focused array) were prepared using l-1,2-dihexadecyl-*sn*-glycero-3-phosphoethanolamine (DHPE (16)) or *N*-aminooxyacetyl-1,2-dihexadecyl-*sn*-glycero-3-phosphoethanolamine (AOPE (17)) as described.

**TABLE 2:**
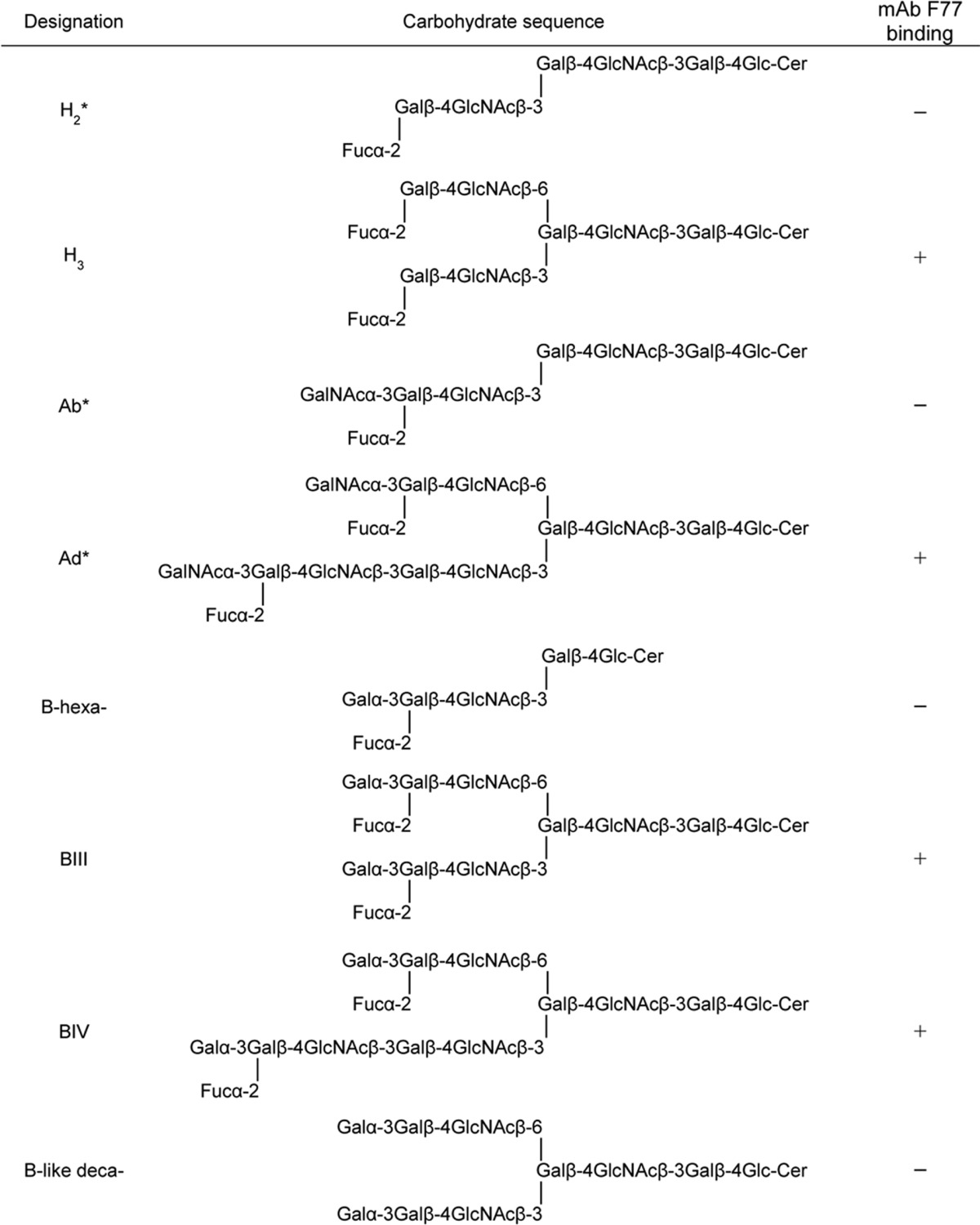
**Blood group H-, A-, and B-active and B-like glycosylceramides and their interactions with mAb F77** Asterisk indicates the glycolipid preparations that contain a minor component with an additional fucose residue detected by MALDI-MS; most likely these are part of the difucosylated Le^y^ sequence.

##### Release and Fractionation of O-Glycans from PSM

*O*-Glycans were released from 800 mg (dry weight) of PSM by alkaline borohydride degradation (18, 19). The products (hexose content: 15 mg as determined by the dot orcinol-sulfuric acid method (16) using galactose as standard) were fractionated by gel filtration using a column (1.6 × 90 cm) of Bio-Gel P4 (Bio-Rad) eluted with H_2_O (Fig. 3*B, inset*), and the pooled fraction a (see Fig. 3*B*) was further fractionated using a column (1.6 × 90 cm) of Bio-Gel P6 (Bio-Rad) eluted with H_2_O (Fig. 3*B*). The selection of pool a for investigation was based on initial antigenic analysis that showed the presence of the F77 antigen activity in fractions eluting from the Bio-Gel P4 column between 4.5 and 8.2 h (data not shown). Seven fractions (*F1–7,* Fig. 3*B*) were collected. Monosaccharide compositions were deduced by matrix-assisted laser desorption/ionization (MALDI) MS. The molar quantities of the oligosaccharides in each fraction were estimated based on the hexose content and molecular mass determined by MALDI-MS.

In small scale experiments, *O*-glycans were nonreductively released from 50 mg (dry weight) of PSM in 10-mg aliquots by hydrazinolysis as described (20). The combined aliquots were fractionated into neutral and acidic components by using a DEAE-Sepharose column (OH form, 1 × 1 cm). The neutral fraction was further purified by a C18 and a graphitized carbon SPE cartridge (Grace Davison Discovery Sciences), yielding 650 μg of hexose. One percent of the released products was labeled with 2-aminobenzamide (2-AB) using a Ludger 2-AB glycan labeling kit as described (20), and 10% (∼100 nmol) was converted to fluorescent NGLs.

##### Generation of Fluorescent NGLs

Fluorescent NGLs were prepared using *N*-aminoacetyl-*N*-(9-anthracenylmethyl)-1,2-dihexadecyl-*sn*-glycero-3-phosphoethanolamine (ADHP) as described (16, 21). In brief, lyophilized reducing oligosaccharides (100 nmol) were mixed with 80 μl of ADHP solution (10 nmol/μl in CHCl_3_/MeOH, 1:1, v/v) and freshly prepared 20 μl of tetrabutylammonium cyanoborohydride (20 μg/μl in MeOH). Reaction was at 60 °C for 16 h. For oligosaccharides larger than hexasaccharide, H_2_O (5%) was included, and incubation was prolonged to 72 h.

Reductively released oligosaccharides (alditols) were conjugated to ADHP after mild periodate oxidation as described (22). In brief, the lyophilized oligosaccharide alditol fractions (∼100 nmol) were mixed with 70 μl of freshly prepared sodium periodate (1.25 mg/ml in imidazole buffer, pH 6.5). The mixture was kept on ice in the dark for 5 min followed by addition of 8 μl of butane-2,3-diol (9 mg/ml) and incubation in the dark for a further 40 min. ADHP solution (200 μl, 10 nmol/μl in CHCl_3_/MeOH, 1:1) and 145 μl of freshly prepared tetrabutylammonium cyanoborohydride (20 μg/μl in MeOH) were then added, and the reaction was carried out at 60 °C for 16 h. Aliquots of the reaction mixtures were analyzed by HPTLC, developed with CHCl_3_/MeOH/H_2_O, 60:35:8 (v/v), and stained with primulin (for detecting lipid) and orcinol (for detecting hexose and fucose) (16).

NGLs were purified using silica cartridges (Waters Sep-Pak) as described (16, 17). The purified NGLs were analyzed by MALDI-MS, quantified on HPTLC by densitometry (21), and stored at −20 °C in CHCl_3_/MeOH/H_2_O, 25:25:8 (v/v).

##### Isolation of Antigen-positive NGLs and Glycolipids

HPTLC plates with aluminum backing were used for isolations of NGLs and glycolipids. Samples were dissolved in CHCl_3_/MeOH/H_2_O, 25:25:8, for application, and plates were developed in CHCl_3_/MeOH/H_2_O, 60:35:8 or 55:45:10. The TLC bands of interest were scraped off the plates, and the NGLs or glycolipids were extracted from the silica gel with CHCl_3_/MeOH/H_2_O, 25:25:8, or eluted off from a mini-column packed with the gel.

HPLC of fluorescent NGLs was carried out on an APS-2 column (5 μm Hypersil, 4.6 × 250 mm) as described (21), using a Gilson system equipped with a fluorescence (λ_ex_ at 255 nm and λ_em_ at 405 nm) and a UV detector (λ_max_ 255 nm). The gradient was CHCl_3_/MeOH/H_2_O, 130:70:9 (solvent A) to 10:20:8 (solvent B), at a flow rate of 0.5 ml/min.

##### Fucosidase Treatment of NGLs and Glycolipids

NGLs or glycolipids (2 nmol) were dissolved in 70 μl of 50 mm sodium citrate buffer, pH 6.0, containing 0.1 mg/ml bovine serum albumin (BSA) and 0.5 mg/ml sodium cholate. After a brief sonication, 200 units of α1–2 fucosidase (New England Biolabs) were added, and the mixture was incubated at 37 °C for 48 h. The reaction mixture was purified by a C18 cartridge, and the products were eluted with CHCl_3_/MeOH/H_2_O, 30:70:30.

##### Mass Spectrometry

MALDI-MS of the oligosaccharides and the derived NGLs was carried out on a TOF Spec-2E (Waters) or an AXIMA Assurance (Shimadzu, Manchester, UK) instrument. Negative-ion MALDI-MS/MS was performed on an AXIMA resonance instrument (Shimadzu, Manchester, UK). For analysis, the oligosaccharides were dissolved in H_2_O and the NGLs in CHCl_3_/MeOH/H_2_O, 25:25:8, at a concentration of 10–20 pmol/μl, and 1 μl was deposited on the sample target together with ∼1 μl of matrix of 2′,4′,6′-trihydroxyacetophenon. Laser energy 20% (coarse) and 60% (fine) was used for the TOF Spec-2E and laser setting 81 for the AXIMA Assurance. For MS/MS, collision gas argon (2 bars) and collision energy 130 V were used.

##### Chromatogram Binding Assays

These were performed essentially as described (16) unless otherwise specified. In brief, NGLs or glycolipids (50–150 pmol) were applied onto the HPTLC plates and developed with the solvent system CHCl_3_/MeOH/0.5 m sodium acetate (25:25:8) for glycolipid extract of PC3 cells, and CHCl_3_/MeOH/H_2_O (60:35:8) for NGLs and human red cell-derived glycolipids. The plates were air-dried and blocked with 3% (w/v) BSA (Sigma A8577) in phosphate/buffered saline (10 mm phosphate, 2.7 mm potassium chloride, and 137 mm sodium chloride, pH 7.4) or HEPES-buffered saline (5 mm HEPES, pH 7.4, 150 mm NaCl, 5 mm CaCl_2_) and overlaid with the following murine mAbs: F77 IgG3 at 20 μg/ml; IgG3 isotype control designated MG3-35 (Abcam) at 20 μg/ml or anti-H type 2 IgG1 designated BRIC231 (Abcam) at 1:100, followed by biotinylated anti-mouse immunoglobulins (Dako) at 1:200 dilution. Binding was detected by overlaying with streptavidin peroxidase (5 μg/ml) followed by color development using 3,3′-diaminobenzidine liquid substrate system (Sigma).

##### Microarray Analyses

For initial screening analysis, microarrays of 492 previously described oligosaccharide probes (23) were used (Glycosciences Array Set 32-39). In the additional microarray set (F77/Ii-focused array), 16 lipid-linked oligosaccharide probes were arrayed in duplicate at 2 and 5 fmol per spot on nitrocellulose-coated glass slides as described (24, 25). A microarray of the 30 mucin-rich glycoproteins was generated with duplicate spots at 30 and 150 pg per spot on nitrocellulose-coated glass slides (designated Mucin Array Set 2).

Microarray analyses were performed essentially as described (23). In brief, after blocking with 3% w/v BSA in HEPES-buffered saline, the arrayed slides were probed with the following murine mAbs: F77 IgG3 and IgG3 control MG3-35, both at 10 μg/ml; anti-blood group A, T36 IgG3 (Abcam) at 1:100 dilution; anti-blood group B antibody designated 89-F (Santa Cruz Biotechnology) at 1 μg/ml; anti-H type 2 at 1:100; anti-H type 1, 17-206 IgG3 (Abcam) at 1:100. mAb binding was detected using biotinylated anti-mouse IgG (Sigma, 1:200) or biotinylated anti-mouse IgM (Sigma, μ-chain-specific, 1:200) followed by an Alexa Fluor-647-labeled streptavidin (Molecular Probes, 1 μg/ml). Binding by the biotinylated *Ulex europaeus* agglutinin, UEA-I (Vector Laboratories), a lectin with blood group H activity, was examined at 50 μg/ml followed by Alexa Fluor-647-labeled streptavidin. Unless otherwise specified, the analyses were performed at 20 °C. Imaging and data analysis were as described (24, 26). Binding signals were probe dose-dependent. Results shown are at 5 fmol/spot for lipid-linked probes and 150 pg per spot for the glycoprotein microarray.

##### Hemagglutination Assays

mAb F77 (2.75 mg/ml) was diluted at 1:500 to 1:20,000 (5.5 μg/ml to 137.5 ng/ml) in 0.9% (w/v) NaCl containing 6% (w/v) human serum albumin. For the hemagglutination gel card column assays (ID-Micro Typing System, Ortho-Clinical Diagnostics, Raritan, NJ), 50 μl of 0.8% suspension of adult red cells of blood groups A, B, or O or cord blood cells of blood group O were mixed with 25 μl of diluted antibody. After incubation at ambient temperature or at 37 °C for 2 min, the gel cards were spun at 90 × *g* in an ID-Micro Typing System centrifuge for 10 min at ambient temperature. The degree of cell agglutination was assessed by the distance of cell sedimentation through the gel and visually scored as 4+, 3+, 2+, and weak. Unagglutinated cells settle at the bottom. End point was taken as 2+.

## RESULTS

**FIGURE 1.**
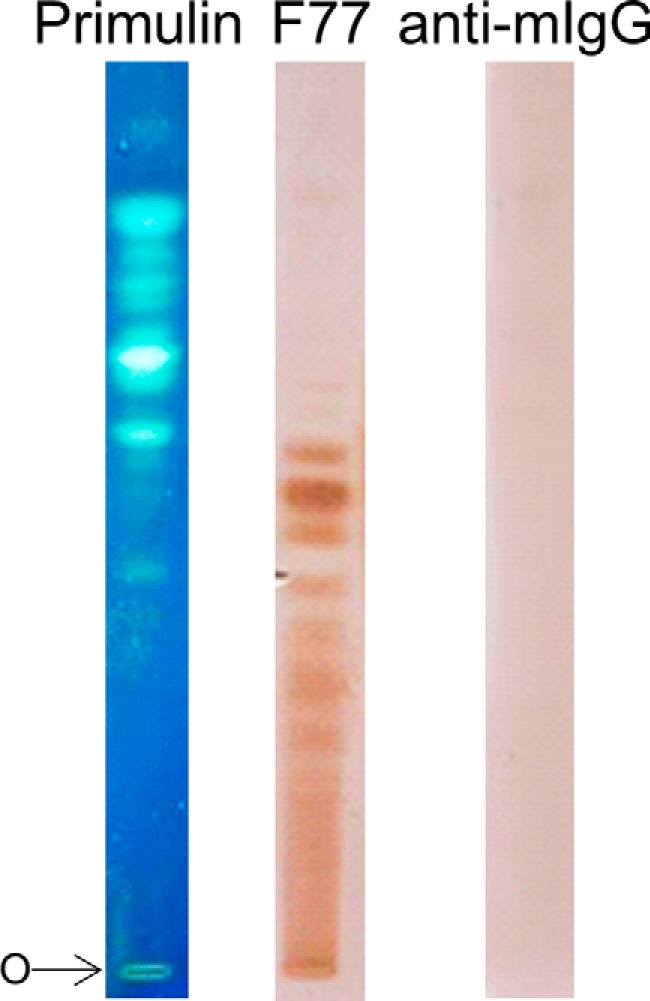
**Binding of mAb F77 to PC3 glycolipid extract resolved by HPTLC.** The PC3 glycolipid extract was chromatographed on a silica gel HPTLC plate using the solvent CHCl_3_/MeOH/0.5 m sodium acetate, 25:25:8 (v/v), and visualized with lipid stain primulin (*left*). Duplicate lanes were overlaid with mAb F77 (*middle*) or with the secondary antibody alone (*right*). Binding was visualized by color development using the streptavidin peroxidase followed by 3,3′-diaminobenzidine liquid substrate system. *O* indicates the origin.

The glycolipid extract from PC3 cells was resolved by HPTLC and stained with primulin to detect the lipid moieties. Numerous primulin-stained components were revealed (Fig. 1). However, only minor components were bound by mAb F77, as indicated by the lack of clear primulin staining corresponding to their positions of migration. As the amounts of glycolipids that can be obtained in PC3 cell extracts are very limited and not readily amenable to detailed characterization, two other approaches were explored toward elucidating the carbohydrate sequence of the F77 antigen. The first approach was to perform microarray analysis with existing sequence-defined oligosaccharide probes. The second approach, based on the fact that *O*-glycans and glycolipids have carbohydrate sequences in common, was microarray analysis with a collection of epithelial mucins as potential large scale sources of the F77 antigen for generating designer arrays.

### 

#### 

##### Unpredicted mAb F77 Binding Detected Two Branched Poly-N-acetyllactosamine-based Blood Group B Glycolipids by Microarray Screening Analysis with Sequence-defined Oligosaccharide Probes

**FIGURE 2.**
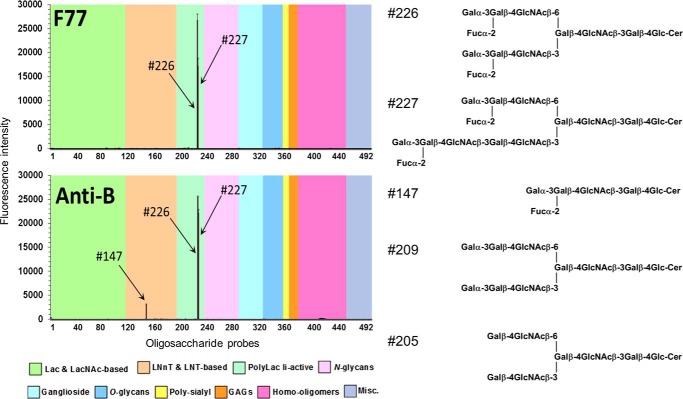
**Microarray analyses of mAbs F77 and anti-B (89-F) with lipid-linked sequence-defined oligosaccharide probes.** The results are the means of fluorescence intensities of duplicate spots, printed at 5 fmol with *error bars* representing half of the difference between the two values. These 492 lipid-linked probes (Glycosciences Laboratory Array Set 32–39) are arranged according to their backbone sequences as annotated in the *colored panels* below the figure. The binding intensities are listed together with the probe sequences in supplemental Table S1.

Microarray analyses of mAb F77 binding using 492 sequence-defined oligosaccharide probes (supplemental Table S1) revealed a strikingly selective binding to two of the probes, 226 and 227 (Fig. 2). These were two branched, poly-*N*-acetyllactosamine-based, blood group B-related glycolipids BIII and BIV, a dodecaglycosyl- and a tetradecaglycosylceramide, respectively, which were also strongly bound by anti-blood group B. In contrast, the unbranched blood group B-related hexaglycosylceramide (probe 147), although bound by anti-blood group B (Fig. 2), gave no detectable binding signal with mAb F77, and binding signals were not detected with the branched B-like analog (probe 209) lacking the α1,2-fucose (13) and the branched I antigen analog, octaglycosylceramide (probe 205) (14), lacking both the α1,2-linked fucose and α1,3-linked galactose. The branched blood group H and A analogs were not available for inclusion in the existing microarray; therefore, it was not possible to assign the epitope to a particular domain on the BIII and BIV glycolipids.

##### PSM Identified as an F77 Antigen-positive Mucin by Microarray Analysis of Several Human and Other Mammalian Mucin-type Glycoproteins

**FIGURE 3.**
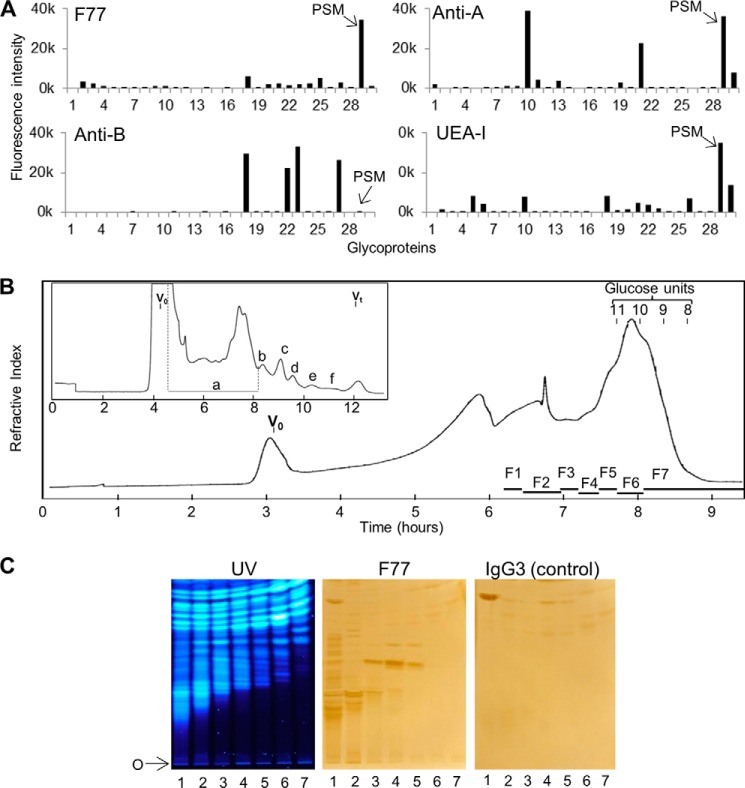
**Detection of F77 antigen on PSM and on minor components among *O*-glycans released from the mucin.** *A,* microarray analyses of mAbs F77, anti-B (89-F), anti-A (T36), and UEA-I lectin with mucin-type glycoproteins. The descriptions of the glycoproteins are in Table 1. Results are the means of fluorescence intensities of duplicate spots printed at 150 pg of glycoprotein per spot. The *error bars* represent half of the difference between the two values. *B,* gel filtration chromatography of the products of reductive alkaline hydrolysis from PSM. *Inset* is the initial chromatography profile using a Bio-Gel P4 column (1.6 × 90 cm) eluted with H_2_O. The *main panel* shows the profile of fraction a from the Bio-Gel P4 column, chromatographed using a Bio-Gel P6 column (1.6 × 90 cm) eluted with H_2_O. *V*_0_ is the void volume of the column; *V_t_* is the total volume; glucose units 8–11 indicate positions of elution of oligosaccharides with degrees of polymerization 8–11 in an acid hydrolysate of dextran. *F1–F7* designate the pooled fractions that were converted to NGLs. *C,* binding of mAb F77 to NGLs derived from the *O*-glycans in fractions 1–7. The NGLs derived from the *O*-glycans in fractions 1–7 were chromatographed on silica gel HPTLC plates, using CHCl_3_/MeOH/H_2_O, 60:35:8 (v/v), as solvent. The fluorescent NGLs were visualized under UV light (*left panel*). The same plate and a duplicate plate were incubated with mAb F77 (*middle panel*) and the isotype IgG3 control, MG3-35 (*right panel*), followed by biotinylated anti-mouse immunoglobulins. Binding was detected as in Fig. 1. *O* indicates the origin.

In the search for an abundant source of F77 antigen, an exploratory microarray analysis of mAb F77 was performed using the 30 preparations of epithelial mucins (Table 1). These were samples that were variously enriched from ovarian cystadenoma fluids, from meconium, or from bovine submaxillary mucin and PSM. Among them were preparations with blood group A, B, or H antigen activities as evidenced by the binding signals they elicited with anti-A, anti-B, or the lectin UEA-I, respectively (Fig. 3*A*). There was little or no binding of mAb F77 to the human glycoproteins included in the array. By far, the highest F77 antigen activity was with PSM (glycoprotein 29, Fig. 3*A*). As predicted (27), strong binding signals were elicited with PSM using anti-A and UEA-I, corresponding to blood group A and H antigen activity, respectively. Blood group B activity was not detected on PSM. Moreover, four strongly B-active glycoproteins (glycoproteins 18, 22, 23, and 27) were only very weakly bound by mAb F77, indicating that the blood group B trisaccharide determinant *per se* is not an essential part of the F77 antigen. Two glycoproteins, 10 and 21, which were strongly A-active and relatively weakly H-active compared with PSM, gave no binding signals with mAb F77. These results suggested that mAb F77 bound to a blood group H-related structural motif present on PSM. Therefore, PSM was selected for further investigation by the designer glycome array approach.

##### F77 Antigen Activity Detected on Minor Components among O-Glycans from PSM

After reductive alkaline hydrolysis of 800 mg of PSM and sequential gel filtration, seven fractions (F1–F7) were obtained (Fig. 3*B*) and analyzed by MALDI-MS. The monosaccharide compositions (in terms of dHex, Hex, and HexNAc) of the major components in each fraction are listed in supplemental Table S2; oligosaccharides identified ranged from larger than undecasaccharides in F1 to pentasaccharides in F7.

Fluorescent NGLs were then prepared after mild periodate oxidation and analyzed by MALDI-MS. The monosaccharide compositions of the major components and the deduced branching positions on the core GalNAcol are in supplemental Table S3, and the chains arising from the 3-linked branches are designated as -OX (OX: -OCH_2_-CH(NHAc)-CH_2_OH-CH_2_-ADHP) and the 6-linked as -OY (OY: -OCH_2_-CH_2_-ADHP).

These highly heterogeneous NGLs in each fraction were first resolved by HPTLC, visualized by UV light, and probed for binding by mAb F77 (Fig. 3*C*). Numerous discrete bands were bound by mAb F77 but not by the IgG3 control. As with the glycolipid extract from PC3 cells (Fig. 1), binding was to minor components among the PSM-derived NGLs that did not correspond to the major fluorescent NGL bands. The NGL fraction 4, which contained the most prominently immunostained band, was selected for further investigation.

##### Isolation of F77 Antigen-positive NGL

**FIGURE 4.**
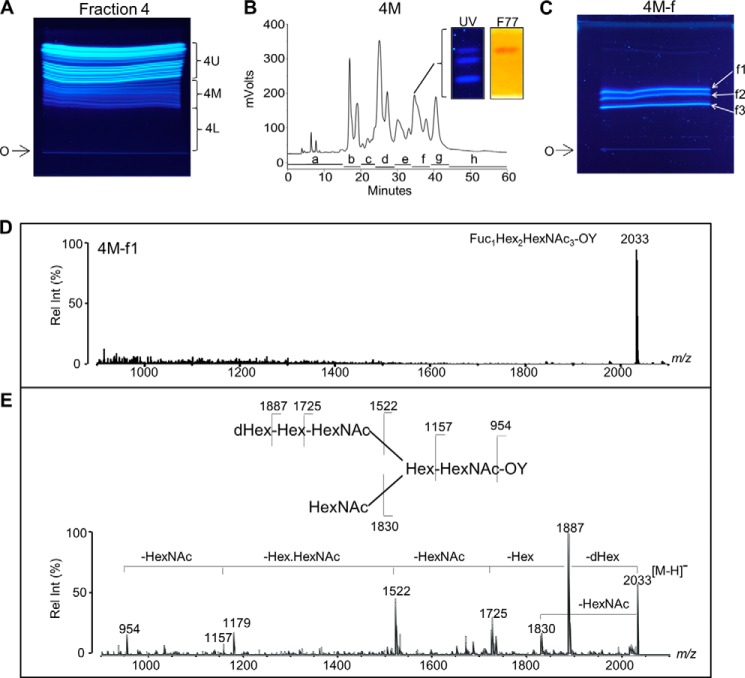
**Isolation and mass spectrometric analysis of an F77 antigen-positive component in fraction 4 of PSM *O*-glycans.** *A,* chromatography of NGLs prepared from fraction 4 (Fig. 3) on HPTLC plates. Three fractions (*upper 4U, middle 4M,* and *lower 4L*) were harvested. *O* indicates origin. *B,* separation of fraction 4M into eight subfractions (*a–h*) by HPLC. *Inset* shows binding of mAb F77 to the upper band in subfraction 4M-f (designated *4M-f1*). *C,* resolution of subfraction 4M-f by HPTLC. The three bands, *4M-f1* to *4M-f3* were harvested. *O* indicates origin. *D,* MALDI-MS analysis of the fraction 4M-f1 in negative mode. The molecular ion [M − H]^−^ at *m*/*z* 2033 indicated the composition shown in the spectrum. *E,* MALDI-CID-MS/MS analysis and the proposed sequence of the F77-positive 4M-f1. *dHex*, deoxyhexose; *Hex*, hexose; *HexNAc*, *N*-acetetylhexosamine; *OY*, 6-linked fragment of core GalNAc after periodate oxidation. *OY*, -OCH_2_-CH_2_-ADHP (22).

The NGLs prepared from fraction 4, although derived from hepta- to octasaccharide alditols (Table 3A), contained mono- to heptasaccharides (Table 3B) after periodate-oxidative splitting of the core GalNAcol before conjugation to ADHP. The main components derived from 3-linked branches (-OX) were di- to hepasaccharides, and those from 6-linked branches (-OY) were mono- to tetrasaccharides. The NGLs of fraction 4 were resolved by semi-preparative HPTLC (Fig. 4*A*), and the region designated 4 m, which includes the F77 antigen-positive component, was harvested and further separated by HPLC (Fig. 4*B*). Eight subfractions, 4M-a to -h, were obtained. F77 antigen activity was monitored using chromatogram-binding assays (data not shown), and one of the subfractions, 4M-f, was found to be F77-positive. The F77 antigen activity was detected in the top band (4M-f1) of the triplet of bands resolved by HPTLC (Fig. 4*B*, *inset*). The 4M-f was resolved into three bands by semi-preparative HPTLC (Fig. 4*C*), and subfractions 4M-f1 to -f3 were harvested.

**TABLE 3:**
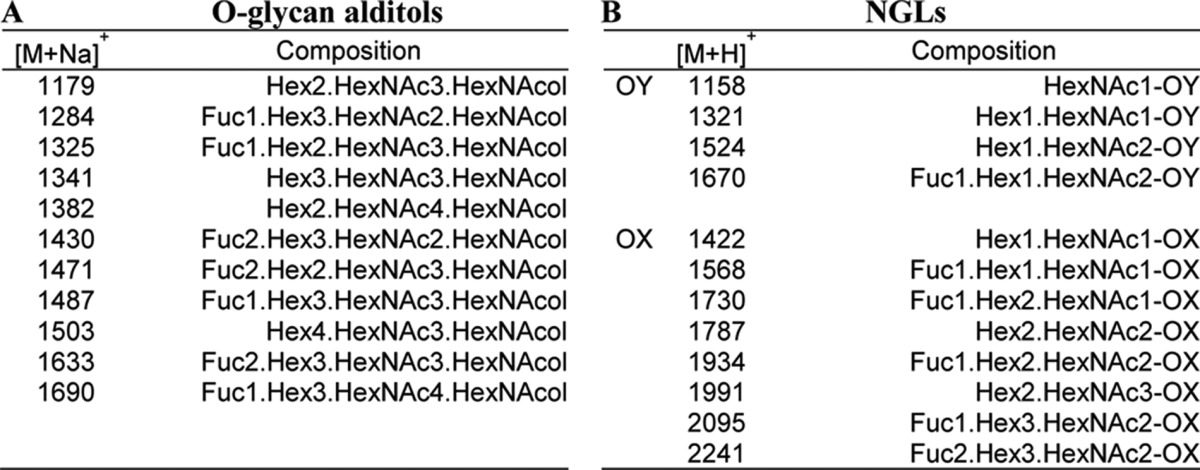
**MALDI-MS and the deduced compositions of the oligosaccharide alditols in Bio-Gel P6 fraction 4 and of the derived NGLs** The abbreviations used are as follows: dHex, deoxyhexose; Hex, hexose; HexNAc, *N*-acetylhexosamine, HexNAcol, *N*-acetylhexosaminitol. -OX and -OY are the 3- and 6-linked fragments of core GalNAc after periodate oxidation; OX: -OCH_2_-CH(NHAc)-CH_2_OH-CH_2_-ADHP; OY: -OCH_2_-CH_2_-ADHP (22).

##### Assignment of the F77 Antigen-positive NGL as a Branched Polylactosamine -based Blood Group H Sequence

NGL subfractions 4M-f1 to -f3 were analyzed by negative ion MALDI-MS. Subfraction 4M-f1 gave a [M − H]^−^ at *m*/*z* 2033 (Fig. 4*D*), corresponding to the NGL of a 6-linked branch with a composition of dHex_1_Hex_2_HexNAc_3_-OY. Subfraction 4M-f2 gave two [M − H]^−^ at *m*/*z* 2240 and 2297, corresponding to the 3-linked branches with compositions of dHex_2_Hex_3_HexNAc_2_-OX and dHex_1_Hex_3_HexNAc_3_-OX, respectively (results not shown). The components in subfraction 4M-f3 gave [M − H]^−^ at *m*/*z* 2094 and 2297, corresponding to dHex_1_Hex_3_HexNAc_2_-OX and dHex_2_Hex_3_HexNAc_2_-OX, respectively (results not shown).

To assign the oligosaccharide sequence of the F77 antigen-positive 4M-f1, MALDI-CID-MS/MS was performed (Fig. 4*E*). Fragment ions *m*/*z* 1887 with neutral loss of dHex (−dHex) from [M − H]^−^ (*m*/*z* 2033) and *m*/*z* 1725 (−dHexHex) and 1522 (−dHexHexHexNAc) identified a sequence of dHex-Hex-HexNAc-. The large gap (365 Da) between fragment ions *m*/*z* 1522 and 1157 corresponded to the loss of HexHexNAc, indicating a branch point as shown in the structure in Fig. 4*E*. The fragment at *m*/*z* 1830 with a loss of HexNAc further indicated a HexNAc branch. Together with the fragment ion *m*/*z* 954 (−dHex from *m*/*z* 1157), these clearly identified an NGL derived from the 6-position of the GalNAcol core with the hexasaccharide sequence shown in Fig. 4*E*.

**FIGURE 5.**
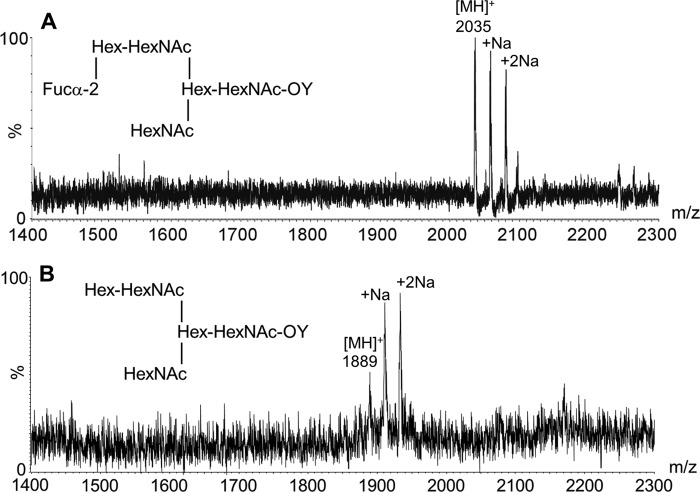
**MALDI-MS analysis of NGL 4M-f1 before (*A*) and after (*B*) treatment with α1–2 fucosidase.** The change in molecular ion [MH]^+^ from *m*/*z* 2035 (*A*) to 1889 (*B*) indicated the capping sequence Fucα1–2Gal in 4M-f1. Abbreviations are as in legend to Fig. 4.

**SCHEME 1:**
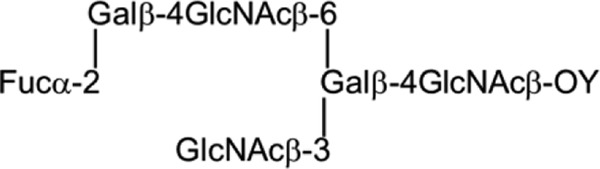


**SCHEME 2:**
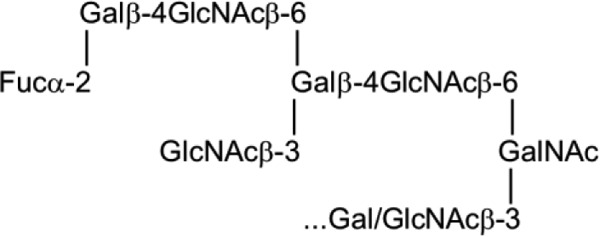


The identity of the dHex in 4M-f1 was assigned by fucosidase treatment and MALDI-MS analysis. The α1–2 fucosidase digestion product gave an MH^+^ at *m*/*z* 1889 (Fig. 5), reduced by 146 Da from the parent NGL 4M-f1 (*m*/*z* 2035), indicating an α1,2-linked fucose. This is in accord with the known blood group H activity in PSM. In separate experiments, 4M-f1 was shown to be bound by a monoclonal anti-H type 2 but not anti-H type 1 (data not shown). Taking into account the F77 antigen activity of the branched blood group B glycolipid BIII and the lack of detection of type 1 backbones in a detailed study of the *O*-glycans of PSM (28), the proposed oligosaccharide sequence of 4M-f1 is a blood group H on a branched backbone of the I-antigen type (29), originating from the C6 position of the core GalNAcol as depicted in Scheme 1. Thus, we propose that the parent *O*-glycan alditol before periodate oxidation was at least the octasaccharide shown in Scheme 2.

**FIGURE 6.**
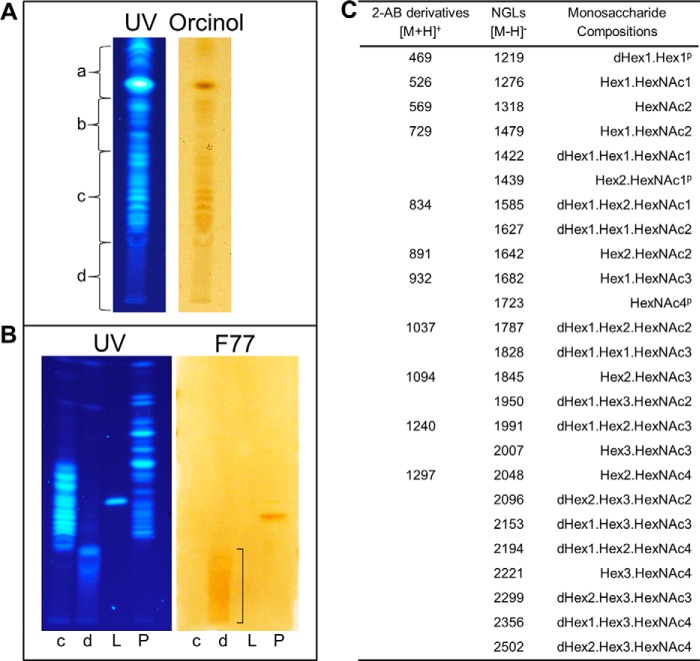
**HPTLC and mAb F77 binding analyses and MALDI-MS of the NGLs and 2-AB derivatives of *O*-glycans nonreductively released from PSM.** *A,* HPTLC analysis of 1% of the NGLs derived from the neutral fraction of *O*-glycans nonreductively released from PSM by hydrazinolysis visualized by UV light (*left panel*) and orcinol staining (*right panel*); *a–d* indicate the positions of migration of fractions harvested by semi-preparative HPTLC of the remaining NGLs. *B,* binding analysis with mAb F77 of NGLs in subfractions c and d, chromatographed together with a fluorescent NGL derived from LnNFPI (included as a molecular weight marker) and NGL fraction 4M referred to in Fig. 4 (*lanes c, d, L,* and *P*, respectively). The fluorescent NGLs were visualized under UV light (*left panel*) and binding by mAb F77 to components in *lanes d*, *bracketed*, and the band corresponding to 4M-f1 in *lane P* (*right panel*) was detected as in legend to Fig. 1. *C,* compositions of 2-AB derivatives and NGLs of *O*-glycans nonreductively released from PSM, deduced from MALDI-MS analysis. The molecular ions of 2-AB derivatives (major components) and those of the corresponding NGLs have been aligned. Abbreviations are as in legend to Fig. 4. *^P^* refers to possible peeled *O*-glycan.

This was corroborated by antigenic analysis of *O*-glycans released nonreductively from PSM in the small scale experiments. An aliquot of the nonreductively released oligosaccharides (65 μg of hexose) was converted into fluorescent NGLs (Fig. 6*A*). Antigenic analysis was carried out with the slow migrating fractions c and d (Fig. 6*B*). A smear of weak immunostaining was detected in faction d, in a region with much slower migration than those of the pentasaccharide LnNFPI-NGL used as reference (*lane L*), and of the immunostained component corresponding to the hexasaccharide-derived NGL, “4M-f1” (*lane P*). The UV fluorescence of the immunostained region in *lane d,* was very faint. As predicted, the positions of migration of these extremely minor and heterogeneous F77 antigen-positive NGLs corresponded to those derived from *O*-glycans that are considerably larger than those detected in faction c, which were hexa- to nonasaccharides as shown by MALDI-MS analysis of both 2-AB and NGL derivatives (Fig. 6*C*).

##### Demonstration of F77 Antigen Expression on Branched Poly-N-acetyllactosamine-based Blood Group H and A Glycolipids

**FIGURE 7.**
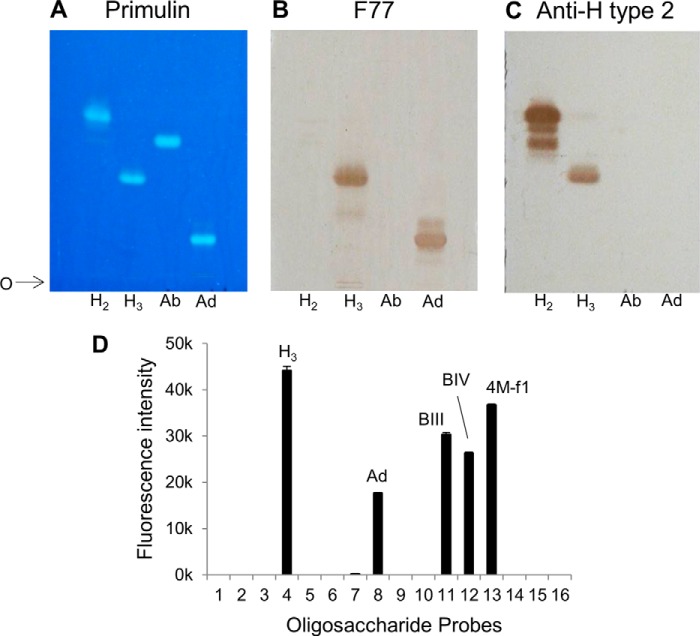
**Demonstration of F77 antigen expression on branched poly-*N*-acetyllactosamine-based blood group H, A, and B glycolipids by chromatogram binding assays and microarray analysis.** *A,* TLC analysis of the four human blood group H- and A-derived glycolipids H_2_, H_3_, Ab, and Ad, revealed by primulin staining for lipid. *B,* binding analysis of mAb F77 using the four blood group H and A glycolipids on a replicate TLC plate to *A. C,* binding analysis of mAb anti-H type 2 (BRIC 231) using the same four blood group H and A glycolipids on a replicate TLC plate to *A*. Solvent used for development is CHCl_3_/MeOH/H_2_O (60:35:8, v/v). *O* indicates the origin. Binding was detected as described in Fig. 1. *D,* microarray analysis of mAb F77, 10 μg/ml, tested at 20 °C with the array of 16 sequence-defined oligosaccharide probes (F77/Ii-focused array). The results are the means of fluorescence intensities of duplicate spots, printed at 5 fmol with error bars representing half of the difference between the two values. The binding intensities are listed together with the probe sequences in supplemental Table S4.

Having identified the red cell-derived branched blood group B glycolipids BIII and BIV and the mucin-derived blood group H-related *O*-glycan sequence as being bound by mAb F77, the question arose as to whether a branched blood group A analog could also be bound by the antibody. To address this, chromatogram binding analyses were performed with the linear and the branched blood group A glycolipids, Ab and Ad, together with the linear and the branched blood group H glycolipids, H_2_ and H_3_, respectively, derived from human red cells (Table 2). The four glycolipid preparations were resolved by HPTLC and visualized by primulin (Fig. 7*A*). Duplicate plates were probed for binding by mAb F77 (Fig. 7*B*) and anti-H type 2 (Fig. 7*C*). The branched H_3_ and Ad but not the linear H_2_ or Ab were bound by mAb F77. The anti-H type 2 antibody bound both to glycolipids H_2_ and H_3_ but not to Ab and Ad, thus ruling out the presence of H type 2-terminating oligosaccharides as contaminants in the preparations of Ab and Ad. Collectively, the results showed that the mAb F77 recognizes the blood group H sequence on a 6-linked branch but can also accommodate an additional α1,3-linked GalNAc or Gal, which constitute the blood group A or B analogs, respectively.

To complement the initial screening microarray analyses, a focused microarray was prepared that included the aforementioned glycan probes with blood group A, B, and H activities based on linear and branched poly-*N*-acetyllactosamine sequences as follows: H_2_, H_3_, Ab, Ad, BIII and BIV, and PSM-4M-f1 (supplemental Table S4). The microarray also included nine additionally generated probes (probes 1, 2, 5, 6, 9, 10, and 14–16) of the blood group H, A, and B series with type 1 and/or type 2 backbones. Among these newly generated probes, the two branched blood group H sequences based on type 1 backbone (probes 14 and 16) gave no binding signals with mAb F77 (Fig. 7*D*). There was also no binding to the branched, difucosylated Le^y^-containing probe 15. Thus, the mAb F77 binding was clearly restricted to glycolipids with the type 2 branched H_3_ (probe 4), Ad (probe 8), BIII and BIV (probes 11 and 12) sequences and the *O*-glycan-derived NGL 4M-f1 (probe 13). The signals elicited by BIII and BIV were lower than H_3_, and the signal with Ad was even lower (Fig. 7*D*).

##### Hemagglutination Properties and Temperature Effects on mAb F77 Binding

As branched poly-*N*-acetyllactosamine sequences (I-antigen type) are predominant on red cells of adult humans, and the linear analogs (i-antigen type) predominate on cord blood cells (30), hemagglutination assays were performed with adult and cord blood cells (results not shown). Indeed, at ambient temperature, when dilutions of mAb F77 were tested with blood group O (H) adult cells and cord blood cells, there was, as predicted, agglutination of both cell types but to a substantially higher titer with the adult cells; the hemagglutination end points of the adult and cord cells were at 78 and 625 ng/ml, respectively. Hemagglutination activity of mAb F77 at 37 °C was lower than at ambient temperature; the end point with group O adult red cells was 156 ng/ml. Thus, mAb F77 had features resembling those of anti-I cold agglutinins (30).

Blood group B and A adult red cells were also agglutinated by mAb F77 but to lower titers with end points of 156 and 313 ng/ml, respectively at ambient temperature. There is also a resemblance here to the so-called anti-HI in normal human sera that have been detected serologically in blood transfusion laboratories; these behave as low titer cold agglutinins, with the hierarchy of titers as follows: adult group O > adult groups A, B, and AB, and ≫ cord blood (31).

**FIGURE 8.**
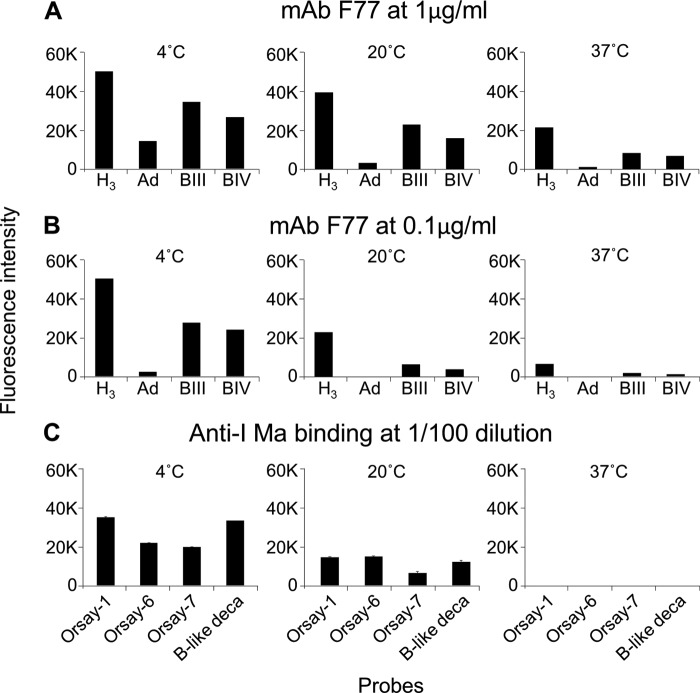
**Temperature dependence of the binding intensities of mAb F77 and anti-I Ma.** *A* and *B,* binding intensities elicited in microarray analyses of mAb F77, 1.0 μg/ml (*A*) and 0.1 μg/ml (*B*) at 4, 20, and 37 °C with the four branched human blood group H, A, and B glycolipids, H_3_, Ad, BIII, and BIV. *C,* binding intensities elicited in microarray analyses of anti-I Ma (1:100) at 4, 20, and 37 °C with NGLs of the chemically synthesized oligosaccharides designated Orsay-1, -6, -7 and the B-like decaosylglyceramide. The results are the means of fluorescence intensities of duplicate spots printed at 5 fmol. The probe sequences of H_3_, Ad, BIII, BIV, and B-like deca- are given in Table 2. The oligosaccharide sequences of Orsay-1, -6, and -7 are as follows: Orsay-1, Galβ-4GlcNAcβ-6Gal; Orsay-6 Galβ-4GlcNAcβ-6(Galβ-4GlcNAcβ-3)Gal; and Orsay-7 Galβ-4GlcNAcβ-6(Galβ-3GlcNAcβ-3)Gal.

The temperature dependence of mAb F77 binding to the branched H, A, and B glycolipids was next investigated at 4, 20, and 37 °C (Fig. 8). The binding intensities were highest at 4 °C, lower at 20 °C, and even lower at 37 °C. The temperature effect was most prominent at 0.1 μg/ml of the antibody (Fig. 8*B*). The temperature dependence of ligand binding was similar to that of the cold agglutinin, anti-I Ma (Fig. 8*C*) (30).

##### Inhibition of F77-PSM Binding by High Concentrations of Short Unbranched A, B, and H Oligosaccharides

Inhibition of binding of mAb F77 to PSM was attempted using free oligosaccharides for the following reasons. First, hemagglutination, although sensitive, is not precisely quantitative and may be influenced by heterogeneities at the red cell surface. Second, antigenic differences may be exaggerated on glycolipids immobilized in the clustered state on matrices. However, only short and unbranched oligosaccharides were available in amounts sufficient for inhibition assays. Nevertheless, inhibitory activities could be recorded at high (millimolar) concentrations. In studies described in the accompanying article (57) in which a monolayer of PC3 cells, which express H but not the A and B antigens, was used as the immobilized substrate, greater inhibition was observed with the H oligosaccharide than the A and B analogs. In this study, where the A- and H-active PSM was used as substrate, the H, A, and B oligosaccharides were equally active as inhibitors of mAb F77 binding (Table 4). These results corroborate the ability of mAb 77 to accommodate the three blood group antigens but with the highest affinity toward the H analog.

**TABLE 4:**
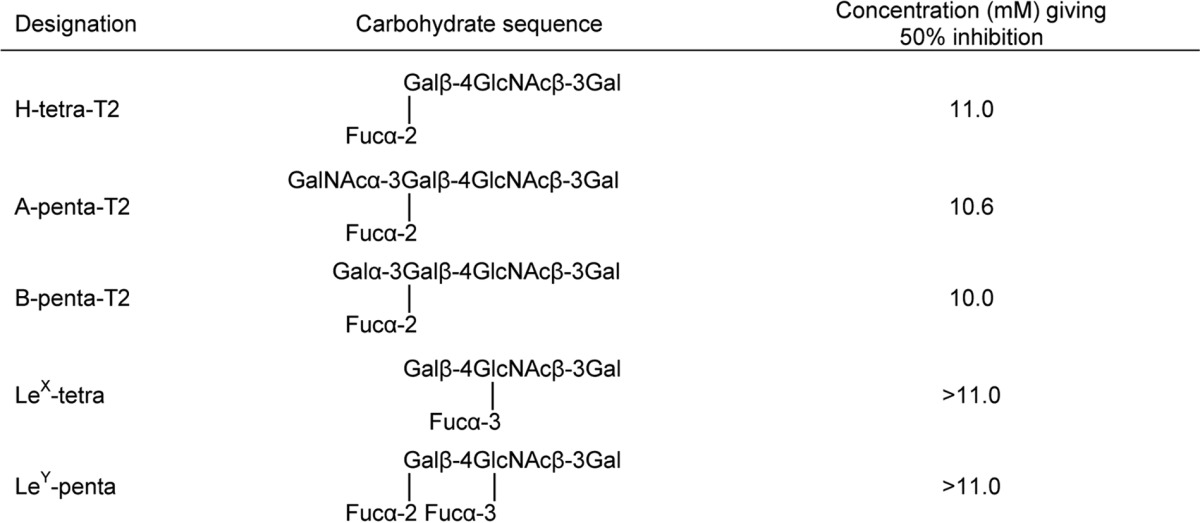
**Inhibition of mAb F77 binding to the A + H antigen-positive PSM by short and unbranched oligosaccharides**

## DISCUSSION

This study illustrates the value of microarrays of sequence-defined glycans in conjunction with glycome-scale designer arrays for the elucidation of novel carbohydrate determinants. The initial microarray analysis of mAb F77 with existing glycan probes gave “hits” with the two blood group B-related glycolipids BIII and BIV. These findings clearly pointed to the presence of the F77 antigen on the two blood group B sequences based on branched poly-*N*-acetyllactosamine backbones. This was quite unpredicted as F77 antigen is known to be widely expressed in prostate cancer tissues (6), although the prevalence of blood group B in Caucasians is only 9% (32). The branched blood group H and A analogs of BIII and BIV glycolipids were not available in the microarray initially. However, the lack of F77 antigen activity on the unbranched analog, the blood group B hexaglycosylceramide, and on an analog of the branched blood group B glycolipids lacking the terminal α1,2-linked blood group H fucose (so called B-like) was an important clue pointing to the presence of the F77 antigen on the branched H, and it was indeed corroborated by the studies that follow.

The F77-active glycolipids of PC3 cells were found to be very minor components within the extremely heterogeneous population. Therefore, the discovery of F77 antigen on an abundantly available mucin, PSM, opened up an alternative route to isolating the antigen, namely by generating designer arrays from the mucin *O*-glycome. Antigenic analyses of the *O*-glycan population released from PSM and arrayed as NGLs led to the isolation and characterization of a F77 antigen-positive component, 4M-f1, as being a blood group H sequence on a 6-linked branch of a di-lactosamine backbone. This assignment is supported by the lack of F77 antigen activity of the two unbranched H-active sequences LnNFPI and H_2_ (*probes 2* and *3* in Fig. 7*D* and supplemental Table S4).

It was pertinent thereafter to evaluate directly the F77 antigen expression on a branched blood group A analog. This was achieved by analysis of the glycolipid Ad, the blood group A analog of BIV, isolated from a glycolipid extract of human red cells. Ad was included in conventional chromatogram-binding analyses and compared with the red cell-derived branched blood group H glycolipid H_3_. In addition, microarray analyses were performed using a newly generated set of probes among which the Ad and H_3_ glycolipids were included in addition to BIII and respective unbranched analogs. It was thus shown that mAb F77 binds to the branched rather than the linear blood group A in addition to the branched B and H on poly-*N*-acetyllactosamine backbones. The agglutination of red cells of A, B, and O types, and more strongly those of adults than of cord blood, were also in accord with the conclusions above that mAb F77 can bind each of the branched A, B, and H antigens. These results are also in complete accord with results of glycosyltransferase transfection studies of Nonaka *et al.* (57).

The approach of generating designer probes (NGLs) from *O*-glycans, following alkaline reductive release from PSM and mild periodate oxidation, had three advantages. First, there is a higher yield of glycans than by nonreductive release (33). Second, there are diagnostic MS features with respect to their origins from 3-linked (OX) or 6-linked (OY) branches at the core GalNAcol residues (34). Third, the cleavage at the core GalNAcol residues gives rise to a population of NGLs with reduced heterogeneity as there arise branches of the same size and sequence from different cores. Thus, the antigen-positive components among the NGLs were resolved into discrete bands on HPTLC (Figs. 3*C* and 4, *A–C*) unlike those derived from nonreductively released glycans that were not only larger but also more heterogeneous (Fig. 6*B*), and they would be difficult to purify in sufficient amounts for detailed analyses. The patterns of binding of F77 to the NGLs from the reductively released *O*-glycans together with the fluorescence labeling of the NGLs facilitated both the visualization and isolation of the main antigen-positive component, 4M-f1.

Thus, our studies establish that F77 antigen can be expressed not only on glycolipids but also on *O*-glycans on glycoproteins. In the previous study (6), there was a lack of a perceptible effect on F77 antigen expression on PC3 and DU 145 cells after treatment with the *O*-glycosylation inhibitor benzyl-α-GalNAc, contrasting with diminished antigen expression after treatment with the glycolipid synthase inhibitor 1-phenyl-2-palmitoylamino-3-morpholino-1-propanol. However, in the accompanying article by Nonaka *et al.* (57), another cell line was tested, namely the F77 antigen-positive 267B1 cell line, co-transfected with *FUT1* and *GCNT3*. Here, treatment with benzyl-α-GalNAc abolished F77 antigen expression. Collectively, these results indicate that there is a predominance of glycolipids with F77 antigen activity in PC3 and DU 145 cells and a low content of *O*-glycosylated proteins relative to those on the transfected 267B1 cells such that the inhibition of *O*-glycan biosynthesis would not have a perceptible effect on expression of the F77 antigen. Recently, more sensitive analyses using immunoprecipitation followed by Western blotting have revealed a glycoprotein that is bound by mAb F77.^8^ The entity of the carrier protein is under investigation.

**SCHEME 3:**
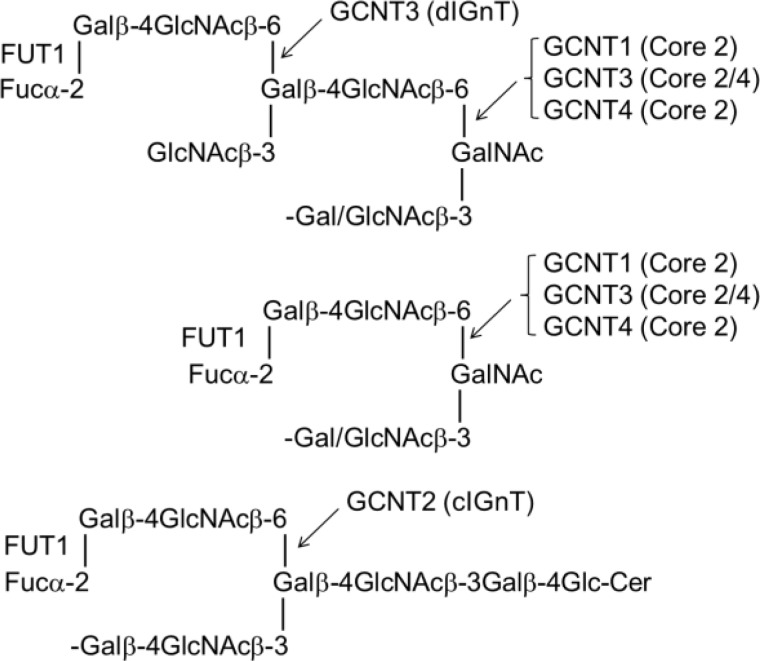


The marked differences in carbohydrate antigen expression on cancer cells compared with normal counterparts have been discussed and reviewed extensively elsewhere (1–4). These arise from diverse changes in carbohydrate chains, including incomplete biosynthesis with loss of capping motifs, exposure of underlying backbones and cores regions, abnormal elongation and branching of backbone regions, inappropriate capping by sialylation, or alterations in sulfation and *neo*synthesis of sequences or overexpression of sequences that are normally minor components for particular cell types (35). These changes are the consequences of multiple factors, including changes in glycosyltransferase and sulfotransferase activities. Highly relevant to F77 antigen expression are α1,2-fucosyltransferases encoded by *FUT1*, which adds α1,2-linked blood group H fucose (36), and β1–6 *N*-acetylglucosaminyltransferase branching enzymes encoded by *GCNTs 1–4*, which synthesize cores 2 and 4 and I-antigen type branched backbones (29, 37), as depicted in Scheme 3.

With the exception of *GCNT3*, the expression of which was found to be down-regulated in colorectal cancer (38), the above-mentioned branching enzyme genes have been observed to be increased in epithelial cancers. Notably, overexpression of *GCNT1* has been reported to be associated with progression of prostate cancer (39, 40). Indeed up-regulation of *GCNT1* has been reported in many cancer types, including endometrial carcinoma (41), breast cancer (42), bladder cancer (43), testicular germ cell tumor (44), and colorectal and pulmonary carcinoma (45, 46). Transfection of this gene into the *GCNT1*-negative prostate carcinoma cell line LNCaP was reported to facilitate adhesion of the cells to type IV collagen and laminin, which may be a factor in aggressive tumor formation (40). Recently, *GCNT2* has been reported to be involved in metastasis of breast cancer through TGF-β signaling, and blocking the expression of *GCNT2* was shown to abrogate the tumor cell migration and invasion (47).

The question then arises as to why F77 antigen that includes a branched backbone sequence is strongly expressed in prostate cancer rather than in other cancers. We propose that the answers lie in the finding that the type 2 blood group H antigen, which is expressed in the normal prostate irrespective of the ABO blood group status, is increased in prostate cancer tissues (48–51). This contrasts with many other cancers in which the branching enzyme genes are overexpressed, but the type 2 H antigen expression is decreased due to α1,3-fucosylation and the formation of the Le^y^ antigen sequence, which we have shown not to be bound by mAb F77 (Fig. 7*D* and supplemental Table S4); alternatively, there may occur competition by sialylation on the terminal galactose instead of α1,2-fucosylation (52). Results of glycosyltransferase gene transfections in the accompanying article (57) are in complete accord both with our findings and our hypothesis. First, expression of F77 antigen was induced in cells lacking the antigen by co-transfection of the gene for α1,2-fucosyltransferase and those for the branching enzymes GCNT1, GCNT2, or GCNT3. Second, RT-PCR analysis revealed the expression of the genes for FUT1, GCNT2, and GCNT3 in F77 antigen-positive cells. Third, siRNA targeting these enzyme genes in antigen-positive cells resulted in significantly reduced levels of the antigen. Although direct uses of mAb F77 in cancer sero-diagnosis and tumor imaging would be precluded because of the expression of the antigen on red cells, one possibility under current investigation is the detection of the antigen on circulating tumor cells. We are also exploring F77 antigen-positive prostatic glycoproteins as potential biomarkers of prostate cancer.

## Supplementary Material

Supplemental Data

## Footnotes

8 Y. Xiao, H. Zhang, and M. Greene, unpublished data.

## Footnotes

7 The abbreviations used are:

HPTLC: high performance thin layer chromatography
ADHP: *N*-aminoacetyl-*N*-(9-anthracenylmethyl)-1,2-dihexadecyl-*sn*-glycero-3-phosphoethanolamine
Hex: hexose
HexNAc: *N*-acetylhexosamine
NGL: neoglycolipid
PSM: porcine stomach mucin
-OX: -OCH_2_-CH(NHAc)-CH_2_OH-CH_2_-ADHP
-OY: -OCH_2_-CH_2_-ADHP
2-AB: 2-aminobenzamide.
