# Supplementary material for: Carbohydrate Sequence of the Prostate Cancer-associated Antigen F77 Assigned by a Mucin O-Glycome Designer Array
Source: J Biol Chem. 2014 Apr 21;289(23):16462–77. doi: 10.1074/jbc.M114.558932 (PMC4047413; doi:10.1074/jbc.M114.558932)
Supplement: Supplemental Data [file supp_289_23_16462__index.html]

Carbohydrate sequence of the prostate cancer-associated antigen F77 assigned by a mucin O-glycome designer array — Carbohydrate Sequence of the Prostate Cancer-associated Antigen F77 Assigned by a Mucin O-Glycome Designer Array — Prostate Cancer-associated Antigen F77 — Supplemental Data 

# Carbohydrate Sequence of the Prostate Cancer-associated Antigen F77 Assigned by a Mucin *O*-Glycome Designer Array

## Supplemental Data

**Files in this Data Supplement:**

- Supplemental tables S1-4 (.pdf, 865 KB) - Supplemental tables S1-4
