# Supplementary material for: Carbohydrate Sequence of the Prostate Cancer-associated Antigen F77 Assigned by a Mucin O-Glycome Designer Array
Source: J Biol Chem. 2014 Apr 21;289(23):16462–77. doi: 10.1074/jbc.M114.558932 (PMC4047413; doi:10.1074/jbc.M114.558932)
Supplement: Supplemental Data [file supp_M114.558932_jbc.M114.558932-1.pdf]

Supplemental Tables S1-4

Carbohydrate sequence of the prostate cancer-associated antigen F77 assigned by a mucin O-glycome designer array

Chao Gao, Yan Liu, Hongtao Zhang, Yibing Zhang, Michiko N. Fukuda, Angelina S. Palma, Radoslaw P. Kozak, Robert A. Childs, Motohiro Nonaka, Zhen Li, Don L. Siegel, Peter Hanfland, Donna M. Peehl, Wengang Chai, Mark I. Greene, Ten Feizi

Supplemental Table S1 **Oligosaccharide probes included in the microarray and the binding signals (means of the fluorescence intensity) elicited with mAb F77 and 89-F anti-B at 5 fmol probe per spot (taken from Fig 2)**

Pos[a]	Probe[b]	Sequence	Binding signals (5fmol/probe spot)	
			**F77**	**Anti-B**
	Lactose and N-acetyllactosamine-based			
1	Galactocerebrosides	Galß-Cer	15	191
2	H-Di	Fucα-2Gal	-[c]	-
3	A-Tri	GalNAcα-3Gal<br>│<br>Fucα-2	-	-
4	B-Tri	Galα-3Gal<br>│<br>Fucα-2	-	3
5	B-Tri-AO	Galα-3Gal-AO<br>│<br>Fucα-2	-	-
6	GSC-426	3-deoxy,3-carboxymethyl-Galß-C30	-	-
7	Sulfatide	SU-3Galβ-Cer	-	-
8	GSF-1	SU-3Galβ-C30	-	-
9	GSC-209	GlcAß-3Galß-Cer42	-	-
10	GSC-210	SU-3GlcAß-3Galß-Cer42	-	-
11	GSC-187	NeuAcα-3Galß-C29	-	-
12	GSC-40	NeuAcα-(S)-3Galß-Cer42	-	-
13	GSC-230	NeuAcα-8NeuAcα-3Galß-Cer36	-	-
14	GSC-27	NeuAcα-6Galß-Cer36	-	-
15	GSC-144	KDNα-6Galß-Cer36	-	-
16	GSC-13	NeuAcα-(S)-6Galß-Cer36	-	-
17	GSC-72	NeuAcα-(S)-6Galβ-(S)-Cer36	-	-
18	GSC-231	NeuAcα-8NeuAcα-6Galß-Cer36	-	-
19	GSC-439	NeuAcα-8NeuAcα-8NeuAcα-6Galß-Cer36	-	-
20	Glucocerebrosides	Glcß-Cer?	-	-
21	GSF-19	SU-6Glcβ-C30	-	-
22	GSC-60	NeuAcα-6Glcß-Cer36	-	-
23	GSC-9	NeuAcα-(S)-6Glcß-Cer36	-	-
24	GSC-62	NeuAcα-2Glcß-Cer36	-	-

25	GSC-59	NeuAcα-6GlcNAcß-Cer36	-	-
26	GSC-95	NeuAcα-(S)-6GlcNAcß-Cer36	-	-
27	GSC-232	NeuAcα-8NeuAcα-6Glcß-Cer36	-	-
28	Lactocerebrosides	Galß-4Glcß-Cer	-	-
29	Lac	Galß-4Glc	-	-
30	Lac-AO	Galß-4Glc-AO	-	-
31	GSC-432	3-deoxy,3-carboxymethyl-Galß-4Glcß-C30	-	-
32	GSC-296	GlcAß-3Galß-4Glcß-C30	-	-
33	GSC-353	SU-3GlcAß-3Galβ-4Glcß-C30	-	-
34	GalNAcα-3Galß-4Glc	GalNAcα-3Galß-4Glc	-	-
35	Globotri-AO	Galα-4Galß-4Glcß-AO	-	-
36	Ceramide trihexoside	Galα-4Galß-4Glcß-Cer	-	-
37	Globoside (P-antigen)	GalNAcß-3Galα-4Galß-4Glcß-Cer	-	-
38	Forssmann glycolipid	GalNAcα-3GalNAcß-3Galα-4Galß-4Glcß-Cer	-	-
39	Fuc(3)-Lac-AO	Galα-4Gal-AO<br>| Fucα-3	-	-
40	GSC-430	3-deoxy,3-carboxymethyl-Galß-3Glcß-C30<br>| Fucα-4	-	-
41	GSC-260	3-deoxy,3-carboxymethyl-Galß-4Glcß-C30<br>| Fucα-3	-	-
42	GSC-150	SU-3Galß-4Glcß-C30<br>| Fucα-3	-	-
43	GSC-160	SU-3Galß-4Glcß-Cer36<br>| Fucα-3	-	-
44	NeuAcα-(3')Lac	NeuAcα-3Galß-4Glc	-	-
45	NeuAcα-(3')Lac-AO	NeuAcα-3Galß-4Glc-AO	-	-
46	Neu4,5Ac-(3')Lac	Neu4,5Acα-3Galß-4Glc	-	-
47	Neu4,5Ac-(3')Lac-AO	Neu4,5Acα-3Galß-4Glc-AO	-	-
48	GSC-16	NeuAcα-3Galß-4Glcß-Cer32	-	-
49	GSC-178	NeuAcα-3Galß-4Glcß-Cer34	-	-
50	GSC-17	NeuAcα-3Galß-4Glcß-Cer36	-	-
51	GSC-18	NeuAcα-3Galß-4Glcß-Cer42	-	-
52	GSC-197	KDNα-3Galβ-4Glcß-Cer28	-	-
53	GSC-199	KDNα-3Galβ-4Glcß-C30	-	-

54	GSC-198	KDNα-3Galβ-4Glcß-Cer34	-	-
55	GSC-75	(4-deoxy)NeuAcα-3Galß-4Glcß-Cer36	-	-
56	GSC-76	(7-deoxy)NeuAcα-3Galß-4Glcß-Cer36	-	-
57	GSC-77	(8-deoxy)NeuAcα-3Galß-4Glcß-Cer36	-	-
58	GSC-153	(4,8-deoxy)NeuAcα-3Galß-4Glcß-Cer36	-	-
59	GSC-51	(9-deoxy)NeuAcα-3Galß-4Glcß-Cer36	-	-
60	GSC-78	(4-OMe)NeuAcα-3Galß-4Glcß-Cer36	-	-
61	GSC-79	(9-OMe)NeuAcα-3Galß-4Glcß-Cer36	-	-
62	GSC-23	(C7)NeuAcα-3Galß1-4Glcß-Cer36	-	-
63	GSC-24	(C8)NeuAcα-3Galß1-4Glcß-Cer36	-	-
64	GSC-50	(C8 diastereoisomer)NeuAcα-3Galß-4Glcß-Cer36	-	-
65	GSC-229	NeuAcα-8NeuAcα-3Galß-4Glcß-Cer36	-	-
66	GSC-96	NeuAcα-9NeuAcα-3Galβ-4Glcß-Cer36	-	-
67	GSC-437	NeuAcα-8NeuAcα-8NeuAcα-3Galβ-4Glcß-Cer36	-	-
68	Neuα-(3')Lac	Neuα-3Galß-4Glc	-	-
69	Neuα-(3')Lac-AO	Neuα-3Galß-4Glc-AO	-	-
70	NeuAcα-(6')Lac	NeuAcα-6Galß-4Glc	-	-
71	NeuAcα-(6')Lac-AO	NeuAcα-6Galß-4Glc-AO	-	-
72	GSC-61	NeuAcα-6Galß-4Glcß-Cer36	-	-
73	GSC-12	NeuAcα-(S)-6Galß-4Glcß-Cer36	-	-
74	GSC-234	NeuAcα-(S)-6Gal(S)β-4Glcß-Cer36	-	-
75	GSC-73	NeuAcα-(S)-6Galβ-4Glcβ-(S)-Cer36	-	-
76	Neuα-(6')Lac	Neuα-6Galß-4Glc	-	-
77	Neuα-(6')Lac-AO	Neuα-6Galß-4Glc-AO	-	-
78	NeuAcß-(3')Lac	NeuAcß-3Galß-4Glc	-	-
79	NeuAcß-(3')Lac-AO	NeuAcß-3Galß-4Glc-AO	-	-
80	NeuAcß-(6')Lac	NeuAcß-6Galß-4Glc	-	-
81	NeuAcß-(6')Lac-AO	NeuAcß-6Galß-4Glc-AO	-	-
82	GSC-161	NeuAcα-3Galß-4Glcß-C30<br>| Fucα-3	-	-
83	GSC-162	NeuAcα-3Galß-4Glcß-Cer36<br>| Fucα-3	-	-
84	LacNAc(1-3)	Galß-3GlcNAc	-	-
85	LacNAc(1-3)-AO	Galß-3GlcNAc-AO	-	-

86	LacNAc	Galß-4GlcNAc	-	-
87	LacNAc-AO	Galß-4GlcNAc-AO	-	-
88	Galα-4Galß-4GlcNAc	Galα-4Galß-4GlcNAc	-	2
89	SU(3')-LN	SU-3Galß-4GlcNAc	-	-
90	Lea-Tri	Galß-3GlcNAc<br>Fucα-4	-	-
91	Lea-Tri-AO	Galß-3GlcNAc-AO<br>Fucα-4	-	-
92	Lex-Tri	Galß-4GlcNAc<br>Fucα-3	-	-
93	Lex-Tri-AO	Galß-4GlcNAc-AO<br>Fucα-3	-	-
94	Lex-Tri-(Me)AO	Galß-4GlcNAc-(Me)AO<br>Fucα-3	-	-
95	SU(3')-Lea-Tri	SU-3Galß-3GlcNAc<br>Fucα-4	-	-
96	SU(3')-Lex-Tri	SU-3Galß-4GlcNAc<br>Fucα-3	-	-
97	NeuAcα-(3')LN1-3	NeuAcα-3Galß-3GlcNAc	-	-
98	NeuAcα-(3')LN1-3-AO	NeuAcα-3Galß-3GlcNAc-AO	-	-
99	NeuAcα-(3')LN	NeuAcα-3Galß-4GlcNAc	-	-
100	NeuAcα-(3')LN-AO	NeuAcα-3Galß-4GlcNAc-AO	17	-
101	PI-1	NeuAcα-3(6-NAc)Galß-4GlcNAc	23	-
102	PI-1-AO	NeuAcα-3(6-NAc)Galß-4GlcNAc-AO	-	-
103	PI-2	NeuAcα-3(6-NBz)Galß-4GlcNAc	-	-
104	PI-2-AO	NeuAcα-3(6-NBz)Galß-4GlcNAc-AO	-	-
105	NeuAcα-(6')LN	NeuAcα-6Galß-4GlcNAc	-	-
106	NeuAcα-(6')LN-AO	NeuAcα-6Galß-4GlcNAc-AO	26	-
107	Neu5,9Ac-(6')LN	Neu5,9Acα-6Galß-4GlcNAc	-	-
108	SA(3')-Lea-Tri	NeuAcα-3Galß-3GlcNAc<br>Fucα-4	-	-
109	SA(3')-Lea-Tri-AO	NeuAcα-3Galß-3GlcNAc-AO<br>Fucα-4	-	-

110	SA(3')-Lex-Tri	NeuAcα-3Galß-4GlcNAc<br>| Fucα-3	-	-
111	SA(3')-Lex-Tri-AO	NeuAcα-3Galß-4GlcNAc-AO<br>| Fucα-3	-	-
112	GSC-440	NeuAcα-3Galß-4GlcNAcß-C30<br>| Fucα-3	-	-
113	GSC-512	Neu4,5Acα-3Galß-4GlcNAcß-C30<br>| Fucα-3	-	-
114	GSC-513	Neu5,9Acα-3Galß-3GlcNAcß-C30<br>| Fucα-4	-	-
115	GSC-511	Neu5,9Acα-3Galß-4GlcNAcß-C30<br>| Fucα-3	-	-
	Lacto-N-neotetraose and Lacto-N-tetraose-based			
116	GSC-225	(3-carboxymethyl)Galß-4GlcNAcß-3Galß-Cer36<br>| Fucα-3	-	-
117	GSC-236	SU3<br>| Galß-4GlcNAcß-3Galß-C30<br>| Fucα-3	-	-
118	GSC-479	NeuAcα-3Galß-4GlcNAcß-3Galß-C30<br>| Fucα-3	-	-
119	GSC-105	NeuAcα-3Galß-4GlcNAcß-3Galß-Cer36<br>| Fucα-3	-	-
120	GSC-121	NeuAcα-3Galß-4GlcNAcß-3Galß-Cer36<br>| (3-deoxy)Fucα-3	-	-
121	GSC-123	NeuAcα-3Galß-4GlcNAcß-3Galß-Cer36<br>| (4-deoxy)Fucα-3	-	-
122	GSC-133	NeuAcα-3Galß-4GlcNAcß-3Galß-Cer36<br>| (2-OMe)Fucα-3	-	-
123	GSC-131	NeuAcα-3Galß-4GlcNAcß-3Galß-Cer36<br>| Quvα-3	-	-

124	GSC-163	NeuAcα-3Galß-4GlcNAcß-3Galß-Cer36<br>│<br>Rhaα-3	3	-
125	GSC-127	NeuAcα-3Galß-4GlcNAcß-3Galß-Cer36<br>│<br>(6-deoxy)L-Talα-3	-	-
126	GSC-341	KDNα-3Galβ-4GlcNAcβ-3Galß-C30<br>│<br>Fucα-3	-	-
127	GSC-177	NeuGcα-3Galβ-4GlcNAcβ-3Galß-Cer36<br>│<br>Fucα-3	-	-
128	GSC-175	NeuAcα-3(4-deoxy)Galß-4GlcNAcß-3Galß-Cer36<br>│<br>Fucα-3	-	-
129	GSC-176	NeuAcα-3(6-deoxy)Galß-4GlcNAcß-3Galß-Cer36<br>│<br>Fucα-3	-	-
130	GSC-257	NeuAcα-3(4,6-deoxy)Galß-4GlcNAcß-3Galß-Cer36<br>│<br>Fucα-3	-	-
131	DLNN	GlcNAcß-3Galß-4Glc	-	-
132	LNT	Galß-3GlcNAcß-3Galß-4Glc	-	-
133	Paragloboside	Galß-4GlcNAcß-3Galß-4Glcß-Cer	-	-
134	LNnT	Galß-4GlcNAcß-3Galß-4Glc	-	-
135	B-like pentaosylceramide	Galα-3Galß-4GlcNAcß-3Galß-4Glcß-Cer	-	-
136	Klaus glycolipid	Galß-3Galß-4GlcNAcß-3Galß-4Glcß-Cer	-	-
137	GSC-207	GlcAß-3Galß-4GlcNAcß-3Galß-4Glcß-C30	-	-
138	GSC-191	GlcAß-3Galß-4GlcNAcß-3Galß-4Glcß-Cer36	-	-
139	GSC-189	GlcAß-3Galß-4GlcNAcß-3Galß-4Glcß-Cer42	-	-
140	SU(3')-Tri	SU-3Galß-4GlcNAcß-3Gal	-	-
141	GSC-208	SU-3GlcAß-3Galß-4GlcNAcß-3Galß-4Glcß-C30	-	-
142	GSC-192	SU-3GlcAß-3Galß-4GlcNAcß-3Galß-4Glcß-Cer36	-	-
143	GSC-190	SU-3GlcAß-3Galß-4GlcNAcß-3Galß-4Glcß-Cer42	-	-
144	Led-II pentaosylceramide	Fucα-2Galß-3GlcNAcß-3Galß-4Glcß-CerA	-	-
145	Led-I pentaosylceramide	Fucα-2Galß-3GlcNAcß-3Galß-4Glcß-CerB	11	-
146	LNFP-I	Fucα-2Galß-3GlcNAcß-3Galß-4Glc	-	-
147	B-hexaosylceramide	Galα-3Galß-4GlcNAcß-3Galß-4Glcß-Cer<br>│<br>Fucα-2	-	2,996

148	A-Hexa	GalNAcα-3Galß-3GlcNAcß-3Galß-4Glc<br>Fucα-2	-	-
149	A-Hepta	GalNAcα-3Galß-3GlcNAcß-3Galß-4Glc<br>Fucα-2 Fucα-4	-	-
150	LNFP-II	Galß-3GlcNAcß-3Galß-4Glc<br>Fucα-4	-	-
151	LNDFH-II	Galß-3GlcNAcß-3Galß-4Glc<br>Fucα-4 Fucα-3	-	-
152	Leb-hexaosylceramide	Fucα-2Galß-3GlcNAcß-3Galß-4Glcß-Cer<br>Fucα-4	-	-
153	LNDFH-I	Fucα-2Galß-3GlcNAcß-3Galß-4Glc<br>Fucα-4	-	-
154	LNTFH-I	Fucα-2Galß-3GlcNAcß-3Galß-4Glc<br>Fucα-4 Fucα-2	-	-
155	LNFP-III	Galß-4GlcNAcß-3Galß-4Glc<br>Fucα-3	-	-
156	LNFP-III-AO	Galß-4GlcNAcß-3Galß-4Glc-AO<br>Fucα-3	-	-
157	LNnDFH-I	Fucα-2Galß-4GlcNAcß-3Galß-4Glc<br>Fucα-3	-	-
158	LNnDFH-II	Galß-4GlcNAcß-3Galß-4Glc<br>Fucα-3 Fucα-3	-	-
159	LNnDFH-V	Galß-4GlcNAcß-3Galß-4Glc<br>Fucα-3 Fucα-2	-	4
160	LNnTFH-I	Fucα-2Galß-4GlcNAcß-3Galß-4Glc<br>Fucα-3 Fucα-2	-	-
161	SU(3')-LNFP-II	SU-3Galß-3GlcNAcß-4Galß-4Glc<br>Fucα-4	-	29
162	SU(6')-LNFP-II	SU-6Galß-3GlcNAcß-3Galß-4Glc<br>Fucα-4	-	-

163	SU(3')-LNFP-III	SU-3Galß-4GlcNAcß-3Galß-4Glc<br>Fucα-3	20	-
164	SU(6')-LNFP-III	SU-6Galß-4GlcNAcß-3Galß-4Glc<br>Fucα-3	-	-
165	SU(3',6)-LNFP-III	SU-6<br>SU-3Galß-4GlcNAcß-3Galß-4Glc<br>Fucα-3	-	-
166	LSTa	NeuAcα-3Galß-3GlcNAcß-3Galß-4Glc	-	-
167	GSC-272	NeuAcα-3Galβ-3GlcNAcβ-3Galβ-4Glcß-C30	-	-
168	GSC-147	KDNα-3Galβ-3GlcNAcβ-3Galβ-4Glcß-Cer36	-	-
169	GSC-396	NeuGcα-3Galβ-3GlcNAcβ-3Galβ-4Glcß-C30	-	-
170	LSTb	Galβ-3GlcNAcβ-3Galβ-4Glc<br>NeuAcα-6	-	19
171	GSC-397	NeuGcα-6Galβ-3GlcNAcβ-3Galβ-4Glcß-C30	-	-
172	DSLNT	NeuAcα-3Galß-3GlcNAcß-3Galß-4Glc<br>NeuAcα-6	4	-
173	Sialylparagloboside	NeuAcα-3Galß-4GlcNAcß-3Galß-4Glcß-Cer	-	-
174	GSC-273	NeuAcα-3Galβ-4GlcNAcβ-3Galβ-4Glcß-C30	-	-
175	GSC-31	NeuAcα-3Galß-4GlcNAcß-3Galß-4Glcß-Cer36	10	-
176	LSTc	NeuAcα-6Galβ4-GlcNAcβ3-Galβ4-Glc	-	-
177	GSC-516B	Neuα-3Galß-4GlcNAcß-3Galß-4Glcß-Cer36<br>SU-6	-	-
178	SA(3/6)LNFP-I	NeuAcα-3/6Galß-3GlcNAcß-3Galß-4Glc<br>Fucα-2	-	-
179	SA(3')-LNFP-II	NeuAcα-3Galß-3GlcNAcß-3Galß-4Glc<br>Fucα-4	-	-
180	SA(6')-LNFP-VI	NeuAcα-6Galß-4GlcNAcß-3Galß-4Glc<br>Fucα-3	-	-
181	GSC-533	NeuAcα-3Galß-4GlcNß-3Galß-4Glcß-Cer36<br>Fucα-3	-	-
182	GSC-64	NeuAcα-3Galß-4GlcNAcß-3Galß-4Glcß-Cer36<br>Fucα-3	-	-

183	SA(3')-LNFP-III	NeuAcα-3Galß-4GlcNAcß-3Galß-4Glc<br>Fucα-3	-	-
184	GSC-472	Neuα-3Galß-4GlcNAcß-3Galß-4Glcß-Cer36<br>Fucα-3	-	-
185	GSC-97	NeuAcα-6Galß-4GlcNAcß-3Galß-4Glcß-Cer36<br>Fucα-3	-	-
186	GSC-314	KDNα-3Galß-4GlcNAcß-3Galß-4Glcß-C30<br>Fucα-3	-	-
187	GSC-149	KDNα-3Galβ-4GlcNAcβ-3Galβ-4Glcß-Cer36<br>Fucα-3	-	-
188	GSC-311	KDNα-3Galß-4GlcNAcß-3Galß-4Glcß-C30<br>Rhaα-3	-	-
189	GSC-268	SU-6<br>NeuAcα-3Galß-4GlcNAcß-3Galß-4Glcß-Cer36<br>Fucα-3	-	-
190	GSC-268 deNAc	SU-6<br>Neuα-3Galß-4GlcNß-3Galß-4Glcß-Cer36<br>Fucα-3	-	-
191	GSC-269	SU-6<br>NeuAcα-3Galß-4GlcNAcß-3Galß-4Glcß-Cer36<br>Fucα-3	-	-
192	GSC-406	SU-6<br>Neuα-3Galß-4GlcNAcß-3Galß-4Glcß-Cer36<br>Fucα-3	-	-
193	GSC-270	SU-6 SU-6<br>NeuAcα-3Galß-4GlcNAcß-3Galß-4Glcß-Cer36<br>Fucα-3	-	-
Polylactosamine Ii-antigen type				
194	pLNH	Galß-3GlcNAcß-3Galß-4GlcNAcß-3Galß-4Glc	-	-
195	pLNnH	Galß-4GlcNAcß-3Galß-4GlcNAcß-3Galß-4Glc	-	-
196	GSC-216	GlcAß-3Galß-4GlcNAcß-3Galß-4GlcNAcß-3Galß-4Glcß-Cer42	-	-

<table>
<tr><td>197</td><td>GSC-217</td><td>SU-3GlcAß-3Galß-4GlcNAcß-3Galß-4GlcNAcß-3Galß-4Glcß-Cer42</td><td>-</td><td>-</td></tr>
<tr><td>198</td><td>GSC-218</td><td>GlcAß-3Galß-4GlcNAcß-3Galß-4GlcNAcß-3Galß-4Glcß-Cer36</td><td>34</td><td>-</td></tr>
<tr><td>199</td><td>GSC-219</td><td>SU-3GlcAß-3Galß-4GlcNAcß-3Galß-4GlcNAcß-3Galß-4Glcß-Cer36</td><td>-</td><td>-</td></tr>
<tr><td>200</td><td>LNH</td><td><pre>
Galß-4GlcNAcß-6
              |
              Galß-4Glc
              |
Galß-3GlcNAcß-3
</pre></td><td>-</td><td>-</td></tr>
<tr><td>201</td><td>iLNO</td><td><pre>
Galß-3GlcNAcß-3Galß-4GlcNAcß-6
                             |
                             Galß-4Glc
                             |
               Galß-3GlcNAcß-3
</pre></td><td>-</td><td>-</td></tr>
<tr><td>202</td><td>LND</td><td><pre>
Galß-4GlcNAcß-6
              |
              Galß-4GlcNAcß-6
              | |
Galß-3GlcNAcß-3   Galß-4Glc
                            |
              Galß-3GlcNAcß-3
</pre></td><td>20</td><td>-</td></tr>
<tr><td>203</td><td>LNnH</td><td><pre>
Galß-4GlcNAcß-6
              |
              Galß-4Glc
              |
Galß-4GlcNAcß-3
</pre></td><td>-</td><td>-</td></tr>
<tr><td>204</td><td>Nonaosylceramide</td><td><pre>
              GlcNAcß-6
                      |
GlcNAcß-6   Galß-4GlcNAcß-3Galß-4Glcß-Cer
        | |
        Galß-4GlcNAcß-3
        |
GlcNAcß-3
</pre></td><td>-</td><td>-</td></tr>
<tr><td>205</td><td>I-octaosylceramide</td><td><pre>
Galß-4GlcNAcß-6
              |
              Galß-4GlcNAcß-3Galß-4Glcß-Cer
              |
Galß-4GlcNAcß-3
</pre></td><td>-</td><td>-</td></tr>
<tr><td>206</td><td>I-dodecaosylceramide</td><td><pre>
              Galß-4GlcNAcß-6
                            |
Galß-4GlcNAcß-6   Galß-4GlcNAcß-3Galß-4Glcß-Cer
              | |
              Galß-4GlcNAcß-3
              |
Galß-4GlcNAcß-3
</pre></td><td>38</td><td>-</td></tr>
</table>

<table>
<tr><td>207</td><td>I-hexadecaosylceramide</td><td><pre>   Galß-4GlcNAcß-6
                                          |
              Galß-4GlcNAcß-6   Galß-4GlcNAcß-3Galß-4Glcß-Cer
                            | |
Galß-4GlcNAcß-6   Galß-4GlcNAcß-3
              | |
              Galß-4GlcNAcß-6
              |
Galß-4GlcNAcß-3</pre></td><td>44</td><td>23</td></tr>
<tr><td>208</td><td>I-eicosaosylceramide</td><td><pre>   Galß-4GlcNAcß-6
                                                        |
                            Galß-4GlcNAcß-6   Galß-4GlcNAcß-3Galß-4Glcß-Cer
                                          | |
              Galß-4GlcNAcß-6   Galß-4GlcNAcß-3
                            | |
Galß-4GlcNAcß-6   Galß-4GlcNAcß-6
              | |
              Galß-4GlcNAcß-3
              |
Galß-4GlcNAcß-3</pre></td><td>47</td><td>-</td></tr>
<tr><td>209</td><td>B-like decaosylceramide</td><td><pre>Galα-3Galß-4GlcNAcß-6
                    |
                    Galß-4GlcNAcß-3Galß-4Glcß-Cer
                    |
Galα-3Galß-4GlcNAcß-3</pre></td><td>-</td><td>-</td></tr>
<tr><td>210</td><td>B-like pentadecaosylceramide</td><td><pre>   Galα-3Galß-4GlcNAcß-6
                                  |
Galα-3Galß-4GlcNAcß-6   Galß-4GlcNAcß-3Galß-4Glcß-Cer
                    | |
                    Galß-4GlcNAcß-3
                    |
Galα-3Galß-4GlcNAcß-3</pre></td><td>-</td><td>-</td></tr>
<tr><td>211</td><td>B-like eicosaosylceramide</td><td><pre>   Galα-3Galß-4GlcNAcß-6
                                                |
              Galα-3Galß-4GlcNAcß-6   Galß-4GlcNAcß-3Galß-4Glcß-Cer
                                  | |
Galα-3Galß-4GlcNAcß-6   Galß-4GlcNAcß-3
                    | |
                    Galß-4GlcNAcß-3
                    |
Galα-3Galß-4GlcNAcß-3</pre></td><td>-</td><td>-</td></tr>
</table>

<table>
<tr><td>212</td><td>B-like pentaeicosaosylceramide</td><td><pre>   Galα-3Galß-4GlcNAcß-6
                                                              |
                            Galα-3Galß-4GlcNAcß-6   Galß-4GlcNAcß-3Galß-4Glcß-Cer
                                                | |
              Galα-3Galß-4GlcNAcß-6   Galß-4GlcNAcß-3
                                  | |
Galα-3Galß-4GlcNAcß-6   Galß-4GlcNAcß-6
                    | |
                    Galß-4GlcNAcß-3
                    |
Galα-3Galß-4GlcNAcß-3</pre></td><td>153</td><td>-</td></tr>
<tr><td>213</td><td>pLNFH-IV</td><td><pre>Galß-3GlcNAcß-3Galß-4GlcNAcß-3Galß-4Glc
                     |
                Fucα-3</pre></td><td>9</td><td>-</td></tr>
<tr><td>214</td><td>DFpLNH-II</td><td><pre>Galß-3GlcNAcß-3Galß-4GlcNAcß-3Galß-4Glc
      | |
 Fucα-4   Fucα-3</pre></td><td>-</td><td>-</td></tr>
<tr><td>215</td><td>TFpLNH-I</td><td><pre>Fucα-2Galß-3GlcNAcß-3Galß-4GlcNAcß-3Galß-4Glc
            | |
       Fucα-4   Fucα-3</pre></td><td>-</td><td>-</td></tr>
<tr><td>216</td><td>MFLNH-III</td><td><pre>Galß-4GlcNAcß-6
      | |
 Fucα-3   Galß-4Glc
              |
Galß-3GlcNAcß-3</pre></td><td>-</td><td>-</td></tr>
<tr><td>217</td><td>DFLNH(b)</td><td><pre> Fucα-3
      |
Galß-4GlcNAcß-6
              |
              Galß-4Glc
              |
Galß-3GlcNAcß-3
      |
 Fucα-4</pre></td><td>-</td><td>-</td></tr>
<tr><td>218</td><td>DFLNH(c)</td><td><pre>   Galß-4GlcNAcß-6
                    |
                    Galß-4Glc
                    |
Fucα-2Galß-3GlcNAcß-3
            |
       Fucα-4</pre></td><td>-</td><td>-</td></tr>
<tr><td>219</td><td>DFLNH(a)</td><td><pre>   Galß-4GlcNAcß-6
            | |
       Fucα-3   Galß-4Glc
                    |
Fucα-2Galß-3GlcNAcß-3</pre></td><td>-</td><td>-</td></tr>
</table>

220	TFLNH	Galß-4GlcNAcß-6<br>Fucα-3 Galß-4Glc<br>Fucα-2Galß-3GlcNAcß-3<br>Fucα-4	-	-
221	MFiLNO-IV	Galß-3GlcNAcß-3Galß-4GlcNAcß-6<br>Fucα-3 Galß-4Glc<br>Galß-3GlcNAcß-3	-	-
222	TFiLNO	Galß-3GlcNAcß-3Galß-4GlcNAcß-6<br>Fucα-4 Fucα-3 Galß-4Glc<br>Galß-3GlcNAcß-3<br>Fucα-4	-	-
223	MFLND	Galß-4GlcNAcß-6<br>Fucα-3 Galß-4GlcNAcß-6<br>Galß-3GlcNAcß-3 Galß-4Glc<br>Galß-3GlcNAcß-3	-	-
224	MFLNnH(a)	Galß-4GlcNAcß-6<br>Fucα-3 Galß-4Glc<br>Galß-4GlcNAcß-3	-	-
225	DFLNnH	Fucα-3<br>Galß-4GlcNAcß-6<br>Galß-4Glc<br>Galß-4GlcNAcß-3<br>Fucα-3	-	-
226	B-III dodecaosylceramide	Galα-3Galß-4GlcNAcß-6<br>Fucα-2 Galß-4GlcNAcß-3Galß-4Glcß-Cer<br>Galα-3Galß-4GlcNAcß-3<br>Fucα-2	26,739	25,500

227	B-IV tetradecaosylceramide	Galα-3Galß-4GlcNAcß-6<br>Fucα-2 Galß-4GlcNAcß-3Galß-4Glcß-Cer<br>Galα-3Galß-4GlcNAcß-3Galß-4GlcNAcß-3<br>Fucα-2	17,201	22,120
228	MSLNH	NeuAcα-6Galß-4GlcNAcß-6<br>Galß-4Glc<br>Galß-3GlcNAcß-3	-	-
229	MSLNnH-I	Galß-4GlcNAcß-6<br>Galß-4Glc<br>NeuAcα-6Galß-3GlcNAcß-3	-	-
230	DSLNnH	NeuAcα-6Galß-4GlcNAcß-6<br>Galß-4Glc<br>NeuAcα-6Galß-4GlcNAcß-3	-	-
231	MSMFLNH	Galß-4GlcNAcß-6<br>Fucα-3 Galß-4Glc<br>NeuAcα-3Galß-3GlcNAcß-3	-	-
232	MFMSLNnH	Galß-4GlcNAcß-6<br>Fucα-3 Galß-4Glc<br>NeuAcα-6Galß-3GlcNAcß-3	-	-
233	GSC-221	NeuAcα-3Galß-4GlcNAcß-3Galß-4GlcNAcß-3Galß-4Glcß-Cer36<br>Fucα-3	-	-
234	GSC-220	NeuAcα-3Galß-4GlcNAcß-3Galß-4GlcNAcß-3Galß-4Glcß-Cer36<br>Fucα-3 Fucα-3	-	-
235	C4U	NeuAcα-3Galβ-4GlcNAcβ-3Galβ-3GlcNAc<br>SU-6 SU-6 SU-6	-	-
N-glycan-related				
236	Man2(α2)	Manα-2Man	-	-
237	Man2(α3)	Manα-3Man	-	-
238	Man2(α6)	Manα-6Man	-	-

239	Man3(α3,α6)	Manα-6Man | Manα-3	21	-
240	Man5(α3,α6)	Manα-6Manα-6Man | | Manα-3 Manα-3	-	-
241	Man1GN1	Manß-4GlcNAc	-	-
242	Man2GN1	Manα-3Manß-4GlcNAc	-	-
243	Man2aGN2	Manα-6Manß-4GlcNAcß-4GlcNAc	-	-
244	Man3GN2	Manα-6 | Manß-4GlcNAcß-4GlcNAc | Manα-3	-	-
245	Man4aGN2	Manα-3Manα-6 | Manß-4GlcNAcß-4GlcNAc | Manα-3	-	-
246	Man4bGN2	Manα-6 | Manα-3Manα-6 | Manß-4GlcNAcß-4GlcNAc	-	-
247	Man5GN2	Manα-6 | Manα-3Manα-6 | Manß-4GlcNAcß-4GlcNAc | Manα-3	-	-
248	Man6GN2	Manα-6 | Manα-3Manα-6 | Manß-4GlcNAcß-4GlcNAc | Manα-2Manα-3	-	-
249	Man7(D1)GN2	Manα-6 | Manα-3Manα-6 | Manß-4GlcNAcß-4GlcNAc | Manα-2Manα-2Manα-3	-	-

<table>
<tr><td>250</td><td>Man7(D1)GN2-AO</td><td>Manα-6
|
Manα-3Manα-6
|
Manß-4GlcNAcß-4GlcNAc-AO
|
Manα-2Manα-2Manα-3</td><td>-</td><td>-</td></tr>
<tr><td>251</td><td>Man7(D3)GN2</td><td>Manα-2Manα-6
|
Manα-3Manα-6
|
Manß-4GlcNAcß-4GlcNAc
|
Manα-2Manα-3</td><td>-</td><td>-</td></tr>
<tr><td>252</td><td>Man8(D1D3)GN2</td><td>Manα-2Manα-6
|
Manα-3Manα-6
|
Manß-4GlcNAcß-4GlcNAc
|
Manα-2Manα-2Manα-3</td><td>-</td><td>-</td></tr>
<tr><td>253</td><td>Man9GN2</td><td>Manα-2Manα-6
|
Manα-2Manα-3Manα-6
|
Manß-4GlcNAcß-4GlcNAc
|
Manα-2Manα-2Manα-3</td><td>-</td><td>-</td></tr>
<tr><td>254</td><td>Man9GN2-AO</td><td>Manα-2Manα-6
|
Manα-2Manα-3Manα-6
|
Manß-4GlcNAcß-4GlcNAc-AO
|
Manα-2Manα-2Manα-3</td><td>-</td><td>-</td></tr>
<tr><td>255</td><td>Glc1Man9GN2</td><td>Manα-2Manα-6
|
Manα-6
| |
Manα-2Manα-3 Manβ-4GlcNAcβ-4GlcNAc
|
Glcα-3Manα-2Manα-2Manα-3</td><td>-</td><td>-</td></tr>
<tr><td>256</td><td>Glc1Man9GN2-AO</td><td>Manα-2Manα-6
|
Manα-6
| |
Manα-2Manα-3 Manβ-4GlcNAcβ-4GlcNAc-AO
|
Glcα-3Manα-2Manα-2Manα-3</td><td>-</td><td>-</td></tr>
</table>

<table>
<tr><td>257</td><td>Glc2Man9GN2-AO</td><td>Manα-2Manα-6
|
Manα-6
| |
Manα-2Manα-3 Manβ-4GlcNAcβ-4GlcNAc-AO
|
Glcα-Glcα-3Manα-2Manα-2Manα-3</td><td>-</td><td>-</td></tr>
<tr><td>258</td><td>Glc2Man7(D1)GN1-AO</td><td>Manα-6
|
Manα-3Manα-6
|
Manß-4GlcNAc-AO
|
Glcα-3Glcα-3Manα-2Manα-2Manα-3</td><td>-</td><td>-</td></tr>
<tr><td>259</td><td>Glc3Man7(D1)GN1-AO</td><td>Manα-6
|
Manα-3Manα-6
|
Manß-4GlcNAc-AO
|
Glcα-2Glcα-3Glcα-3Manα-2Manα-2Manα-3</td><td>-</td><td>-</td></tr>
<tr><td>260</td><td>Man3XylGN2</td><td>Manα-6
|
Xylß-2Manß-4GlcNAcß-4GlcNAc
|
Manα-3</td><td>-</td><td>-</td></tr>
<tr><td>261</td><td>N1</td><td>Galß-4GlcNAcß-2Manα-6 Fucα-6
| |
Manß-4GlcNAcß-4GlcNAc
|
Manα-3</td><td>-</td><td>-</td></tr>
<tr><td>262</td><td>N2</td><td>Manα-6
|
Manß-4GlcNAcß-4GlcNAc
|
Galß-4GlcNAcß-2Manα-3</td><td>-</td><td>-</td></tr>
<tr><td>263</td><td>N4</td><td>Galß-4GlcNAcß-2Manα-6
|
Manß-4GlcNAcß-4GlcNAc ?
|
Manα-3</td><td>-</td><td>-</td></tr>
<tr><td>264</td><td>GlcNac2Man3-AO</td><td>GlcNAcß-2Manα-6
|
Man-AO
|
GlcNAcß-2Manα-3</td><td>-</td><td>-</td></tr>
</table>

265	N3	(Galß-4) GlcNAcß-2Manα-6 Fucα-6<br>Manß-4GlcNAcß-4GlcNAc<br>? GlcNAcß-2Manα-3	-	-
266	NGA2	GlcNAcß-2Manα-6<br>Manß-4GlcNAcß-4GlcNAc<br>GlcNAcß-2Manα-3	-	-
267	NGA2B	GlcNAcß-2Manα-6<br>GlcNAcß-4Manß-4GlcNAcß-4GlcNAc<br>GlcNAcß-2Manα-3	-	-
268	NGA3B	GlcNAcß-2Manα-6<br>GlcNAcß-4Manß-4GlcNAcß-4GlcNAc<br>GlcNAcß-4Manα-3<br>GlcNAcß-2	-	-
269	NGA4	GlcNAcß-6<br>GlcNAcß-2Manα-6<br>Manß-4GlcNAcß-4GlcNAc<br>GlcNAcß-2Manα-3<br>GlcNAcß-4	-	-
270	NGA5B	GlcNAcß-2<br>GlcNAcß-4Manα-6<br>GlcNAcß-6<br>GlcNAcß-4Manß-4GlcNAcß-4GlcNAc<br>GlcNAcß-4Manα-3<br>GlcNAcß-2	-	-
271	GNMan5BGN2	Manα-6<br>Manα-3Manα-6<br>GlcNAcß-4Manß-4GlcNAcß-4GlcNAc<br>GlcNAcß-2Manα-3	-	-

<table>
<tr><td>272</td><td>NA2</td><td>Galß-4GlcNAcß-2Manα-6
Manß-4GlcNAcß-4GlcNAc
Galß-4GlcNAcß-2Manα-3</td><td>-</td><td>-</td></tr>
<tr><td>273</td><td>NA3</td><td>Galß-4GlcNAcß-2Manα-6
Manß-4GlcNAcß-4GlcNAc
Galß-4GlcNAcß-4Manα-3
Galß-4GlcNAcß-2</td><td>-</td><td>-</td></tr>
<tr><td>274</td><td>NA4</td><td>Galß-4GlcNAcß-6
Galß-4GlcNAcß-2Manα-6
Manß-4GlcNAcß-4GlcNAc
Galß-4GlcNAcß-4Manα-3
Galß-4GlcNAcß-2</td><td>-</td><td>-</td></tr>
<tr><td>275</td><td>Fuc-GlcNAc</td><td>Fucα-6GlcNAc</td><td>-</td><td>-</td></tr>
<tr><td>276</td><td>Man3FGN2</td><td>Manα-6 Fucα-6
Manß-4GlcNAcß-4GlcNAc
Manα-3</td><td>-</td><td>-</td></tr>
<tr><td>277</td><td>Man3FXylGN2</td><td>Manα-6
Xylβ-2Manα-4GlcNAcβ-4GlcNAc
Manα-3 Fucα-3</td><td>-</td><td>-</td></tr>
<tr><td>278</td><td>NGA2F</td><td>GlcNAcß-2Manα-6 Fucα-6
Manß-4GlcNAcß-4GlcNAc
GlcNAcß-2Manα-3</td><td>-</td><td>-</td></tr>
<tr><td>279</td><td>NA2F</td><td>Galß-4GlcNAcß-2Manα-6 Fucα-6
Manß-4GlcNAcß-4GlcNAc
Galß-4GlcNAcß-2Manα-3</td><td>-</td><td>-</td></tr>
<tr><td>280</td><td>NA2F-AO</td><td>Galß-4GlcNAcß-2Manα-6 Fucα-6
Manß-4GlcNAcß-4GlcNAc-AO
Galß-4GlcNAcß-2Manα-3</td><td>-</td><td>-</td></tr>
</table>

<table>
<tr><td>281</td><td>NA2FB</td><td>Galß-4GlcNAcß-2Manα-6 Fucα-6
| |
GlcNAcß-4Manß-4GlcNAcß-4GlcNAc
|
Galß-4GlcNAcß-2Manα-3</td><td>-</td><td>-</td></tr>
<tr><td>282</td><td>NA3-Lex</td><td>Galß-4GlcNAcß-2Manα-6
|
Manß-4GlcNAcß-4GlcNAc
+Fucα-3 |
Galß-4GlcNAcß-4Manα-3
|
Galß-4GlcNAcß-2</td><td>-</td><td>-</td></tr>
<tr><td>283</td><td>A2(2-6)</td><td>NeuAcα-6Galß-4GlcNAcß-2Manα-6
|
Manß-4GlcNAcß-4GlcNAc
|
NeuAcα-6Galß-4GlcNAcß-2Manα-3</td><td>-</td><td>-</td></tr>
<tr><td>284</td><td>AGP-Bi-Ac2</td><td>NeuAcα-Galß-4GlcNAcß-2Manα-6
|
Manß-4GlcNAcß-4GlcNAc
|
NeuAcα-Galß-4GlcNAcß-2Manα-3</td><td>-</td><td>-</td></tr>
<tr><td>285</td><td>AGP-Bi-Gc2</td><td>NeuGcα-Galß-4GlcNAcß-2Manα-6
|
Manß-4GlcNAcß-4GlcNAc
|
NeuGcα-Galß-4GlcNAcß-2Manα-3</td><td>-</td><td>-</td></tr>
<tr><td>286</td><td>AGP-Bi-AcGc</td><td>NeuGcα-Galß-4GlcNAcß-2Manα-6
? |
Manß-4GlcNAcß-4GlcNAc
|
NeuAcα-Galß-4GlcNAcß-2Manα-3</td><td>-</td><td>-</td></tr>
<tr><td>287</td><td>A3</td><td>NeuAcα-3Galß-4GlcNAcß-2Manα-6
|
Manß-4GlcNAcß-4GlcNAc
|
NeuAcα-3Galß-4GlcNAcß-4Manα-3
|
NeuAcα-6Galß-4GlcNAcß-2</td><td>-</td><td>-</td></tr>
<tr><td>288</td><td>A2F(2-3)</td><td>NeuAcα-3Galß-4GlcNAcß-2Manα-6 Fucα-6
| |
Manß-4GlcNAcß-4GlcNAc
|
NeuAcα-3Galß-4GlcNAcß-2Manα-3</td><td>-</td><td>-</td></tr>
<tr><td colspan="5">Ganglioside-related</td></tr>
<tr><td>289</td><td>GM4</td><td>NeuAcα-3Galβ-Cer</td><td>-</td><td>-</td></tr>
<tr><td>290</td><td>SM3</td><td>SU-3Galβ-4Glcß-Cer</td><td>16</td><td>-</td></tr>
<tr><td>291</td><td>Haematoside</td><td>NeuAcα-3Galβ-4Glcß-Cer</td><td>-</td><td>-</td></tr>
</table>

292	GM3	NeuAcα-3Galß-4Glcß-Cer	-	-
293	GM3(Gc)	NeuGcα-3Galß-4Glc-Cer	-	-
294	Asialo-GM2	GalNAcβ-4Galβ-4Glcβ-Cer	-	-
295	SM2	GalNAcß-4Galß-4Glcß-Cer<br>SU-3	-	-
296	SB2	SU-3GalNAcß-4Galß-4Glcß-Cer<br>SU-3	-	-
297	GM2	GalNAcβ-4Galβ-4Glcβ-Cer<br>NeuAcα-3	-	-
298	GSC-576	GalNAcβ-4Galβ-3Glcβ-C30<br>NeuAcα-3	-	-
299	GSC-108	GalNAcβ-4Galβ-4Glcβ-Cer36<br>NeuAcα-3	-	-
300	GSC-193	GalNAcβ-4Galβ-4Glcβ-Cer36<br>KDNα-3	-	-
301	Asialo-GM1	Galβ-3GalNAcβ-4Galβ-4Glcβ-Cer	-	-
302	Asialo-GM1-Tetra	Galß-3GalNAcß-4Galß-4Glc	-	-
303	SM1a	Galß-3GalNAcß-4Galß-4Glcß-Cer<br>SU-3	-	-
304	SB1a	SU-3Galß-3GalNAcß-4Galß-4Glcß-Cer<br>SU-3	-	-
305	GSC-335	SU-6<br>NeuAcα-3Galß-3GalNAcß-4Galß-4Glcß-Cer36	-	-
306	GM1	Galβ-3GalNAcβ-4Galβ-4Glcβ-Cer<br>NeuAcα-3	-	-
307	GM1-penta	Galβ-3GalNAcβ-4Galβ-4Glc<br>NeuAcα-3	-	-
308	GM1(Gc)	Galβ-3GalNAcβ-4Galβ-4Glcβ-Cer<br>NeuGcα-3	-	-
309	GM1(Gc)-penta	Galβ-3GalNAcβ-4Galβ-4Glc<br>NeuGcα-3	-	-

310	GD1a	NeuAcα-3Galß-3GalNAcß-4Galß-4Glcß-Cer<br>| NeuAcα-3	-	-
311	GD1a-hexa	NeuAcα-3Galß-3GalNAcß-4Galß-4Glc<br>| NeuAcα-3	-	-
312	GalNAc-GD1a(Ac,Gc)	GalNAcß-4Galß-3GalNAcß-4Galß-4Glcß-Cer<br>| | NeuGcα-3 NeuAcα-3<br><br>GalNAcß-4Galß-3GalNAcß-4Galß-4Glcß-Cer<br>| | NeuAcα-3 NeuGcα-3	-	-
313	GSC-195	KDNα-3Galβ-3GalNAcβ-4Galβ-4Glcß-Cer36<br>| KDNα-3	-	-
314	GD3	NeuAcα-8NeuAcα-3Galß-4Glcß-Cer	-	-
315	GD3-tetra	NeuAcα-8NeuAcα-3Galß-4Glc	-	-
316	GD3-tetra-AO	NeuAcα-8NeuAcα-3Galß-4Glc-AO	-	-
317	GD2	GalNAcß-4Galß-4Glcß-Cer<br>| NeuAcα-8NeuAcα-3	-	-
318	GD1b	Galß-3GalNAcß-4Galß-4Glcß-Cer<br>| NeuAcα-8NeuAcα-3	-	-
319	GT1a	NeuAcα-8NeuAcα-3Galβ-3GalNAcβ-4Galβ-4Glcβ-Cer<br>| NeuAcα-3	-	-
320	GT1b	NeuAcα-3Galβ-3GalNAcβ-4Galβ-4Glcβ-Cer<br>| NeuAcα-8NeuAcα-3	-	-
321	GQ1b	NeuAcα-8NeuAcα-3Galβ-3GalNAcβ-4Galβ-4Glcβ-Cer<br>| NeuAcα-8NeuAcα-3	-	-
322	GSC-442	GalNAcβ-4Galβ-4Glcß-Cer36<br>| NeuAcα-6	-	-
323	GSC-68	NeuAcα-6Galß-3GalNAcß-4Galß-4Glcß-Cer36	-	-
324	GSC-107	NeuAcα-6Galβ-3GalNAcβ-4Galβ-4Glcß-Cer36<br>| NeuAcα-6	-	-
325	GSC-118	NeuAcα-3Galß-3GalNAcβ-4Galβ-4Glcß-Cer36<br>| NeuAcα-6	-	-
	O-glycan related			

326	GalNAc-Ser	GalNAc-Ser	-	-
327	GalNAc-Thr	GalNAc-Thr	-	-
328	BSM-Di-A1-AO	NeuGcα-6GalNAc-AO	-	-
329	BSM-Di-A2-AO	NeuAcα-6GalNAc-AO	-	-
330	GalNAcα-3GalNAc	GalNAcα-3GalNAc	-	-
331	Galß-3GalNAc	Galß-3GalNAc	-	-
332	Galß-3GalNAc-AO	Galß-3GalNAc-AO	-	-
333	Galß-6GalNAc	Galß-6GalNAc	8	-
334	Galß-6GalNAc-AO	Galß-6GalNAc-AO	-	-
335	Man-Ser	Manα-Ser	-	-
336	Man-Ser-Succ	Man-Ser-Succ	-	-
337	Man-Thr	Man-Thr	15	-
338	Man-Thr-Succ	Man-Thr-Succ	-	-
339	A8/1	GlcNAcα-4Galβ-OX	-	-
340	A8/2	SU-6<br>| Fucα-3GlcNAcβ-OY	-	-
341	A15/1	SU-6GlcNAcß-OY	-	-
342	A15/3	GlcNAcα-4Galβ-3Galβ-OX<br>| Fucα-2	-	-
343	B13/a	GlcAβ-3Galβ-3GlcNAcβ-OX	-	-
344	Notch-1	Fucα-Thr	-	-
345	Notch-2	GlcNAcβ-3Fucα-Thr	-	-
346	Notch-3	Galβ-4GlcNAcβ-3Fucα-Thr	-	3
347	GSC-488	NeuAcα-3Galß-3GalNAcß-C30	-	-
348	GSC-491	NeuAcα-3Galß-3(6-deoxy-6-CH2COOH)GalNAcß-C30	-	-
349	GSC-489	SU-6<br>| NeuAcα-3Galß-3GalNAcß-C30	-	-
350	DST	NeuAcα-3Galß-3GalNAc<br>| NeuAcα-6	-	-
351	DST-AO	NeuAcα-3Galß-3GalNAc-AO<br>| NeuAcα-6	23	-
352	GSC-490	NeuAcα-3Galß-3GalNAcß-C30<br>| NeuAcα-6	-	-
353	GlcNAcß-3Fuc-AO	GlcNAcß-3Fuc-AO	-	-

<table>
<tr><td>354</td><td>GlcNAcß-2Man-AO</td><td>GlcNAcß-2Man-AO</td><td>-</td><td>-</td></tr>
<tr><td></td><td colspan="4">Polysialyl</td></tr>
<tr><td>355</td><td>SA2(α8)</td><td>NeuAcα-8NeuAc</td><td>-</td><td>-</td></tr>
<tr><td>356</td><td>SA3(α8)</td><td>NeuAcα-8NeuAcα-8NeuAc</td><td>-</td><td>-</td></tr>
<tr><td>357</td><td>SA4(α8)</td><td>NeuAcα-8NeuAcα-8NeuAcα-8NeuAc</td><td>-</td><td>-</td></tr>
<tr><td>358</td><td>SA5(α8)</td><td>NeuAcα-8NeuAcα-8NeuAcα-8NeuAcα-8NeuAc*</td><td>-</td><td>-</td></tr>
<tr><td>359</td><td>SA6(α8)</td><td>NeuAcα-8NeuAcα-8NeuAcα-8NeuAcα-8NeuAcα-8NeuAc*</td><td>-</td><td>-</td></tr>
<tr><td>360</td><td>SA7(α8)</td><td>NeuAcα-8NeuAcα-8NeuAcα-8NeuAcα-8NeuAcα-8NeuAcα-8NeuAc*</td><td>-</td><td>-</td></tr>
<tr><td>361</td><td>SA8(α8)</td><td>NeuAcα-8NeuAcα-8NeuAcα-8NeuAcα-8NeuAcα-8NeuAc-8NeuAcα-8NeuAc*</td><td>-</td><td>-</td></tr>
<tr><td>362</td><td>SA9(α8)</td><td>NeuAcα-8NeuAcα-8NeuAcα-8NeuAcα-8NeuAcα-8NeuAc-8NeuAcα-8NeuAc-8NeuAcα*</td><td>-</td><td>-</td></tr>
<tr><td>363</td><td>SA10(α8)</td><td>NeuAcα-8NeuAcα-8NeuAcα-8NeuAcα-8NeuAcα-8NeuAc-8NeuAcα-8NeuAcα-8NeuAcα-8NeuAc*</td><td>-</td><td>-</td></tr>
<tr><td></td><td colspan="4">Glycosaminoglycan-related</td></tr>
<tr><td>364</td><td>HA-S4</td><td>GlcAß-3GlcNAcß-4GlcAß-3GlcNA*</td><td>34</td><td>-</td></tr>
<tr><td>365</td><td>HA-S14</td><td>GlcAß-3GlcNAcß-4GlcAß-3GlcNAcß-4GlcAß-3GlcNAcß-4GlcAß-3GlcNAcß-4GlcAß-3GlcNAcß-4GlcAß-3GlcNAcß-
4GlcAß-3GlcNA*</td><td>-</td><td>-</td></tr>
<tr><td>366</td><td>Hep-Di IS</td><td>ΔUA-4GlcNS
| |
SU-2 6-SU</td><td>-</td><td>-</td></tr>
<tr><td>367</td><td>Hep-Di-IS-AO</td><td>ΔUA-4GlcNS-AO
| |
SU-2 6-SU</td><td>-</td><td>-</td></tr>
<tr><td>368</td><td>CSA-4</td><td>ΔUA-3GalNAcß-4GlcAß-3GalNAc*
| |
SU-4 SU-4</td><td>-</td><td>-</td></tr>
<tr><td>369</td><td>CSA-14</td><td>ΔUA-3GalNAcß-4GlcAß-3GalNAcß-4GlcAß-3GalNAcß-4GlcAß-3GalNAcß-4GlcAß-3GalNAcß-4GlcAß-
| | | | |
SU-4 SU-4 SU-4 SU-4 SU-4

-3GalNAcß-4GlcAß-3GalNAc*
| |
SU-4 SU-4</td><td>-</td><td>-</td></tr>
<tr><td>370</td><td>CSB-4</td><td>ΔUA-3GalNAcß-4IdoAα-3GalNAc*
| |
SU-4 SU-4</td><td>-</td><td>-</td></tr>
<tr><td>371</td><td>CSB-14</td><td>ΔUA-3GalNAcß-4IdoAα-3GalNAcß-4IdoAα-3GalNAcß-4IdoAα-3GalNAcß-4IdoAα-3GalNAcß-4IdoAα-
| | | | |
SU-4 SU-4 SU-4 SU-4 SU-4

-3GalNAcß-4IdoAα-3GalNAc*
| |
SU-4 SU-4</td><td>-</td><td>-</td></tr>
</table>

372	CSC-4	ΔUA-3GalNAcß-4GlcAß-3GalNAc*<br>SU-6 SU-6	-	-
373	CSC-14	ΔUA-3GalNAcß-4GlcAß-3GalNAcß-4GlcAß-3GalNAcß-4GlcAß-3GalNAcß-4GlcAß-3GalNAcß-4GlcAß-<br>SU-6 SU-6 SU-6 SU-6 SU-6<br>-3GalNAcß-4GlcAß-3GalNAc*<br>SU-6 SU-6	-	-
374	Hep-4-AO	ΔUA-4GlcNSα-4IdoAα-4GlcNS-AO*<br>SU-2 6-SU SU-2 SU-6	-	-
375	Hep-14-AO	ΔUA-4GlcNSα-4IdoAα-4GlcNSα-4IdoAα-4GlcNSα-4IdoAα-4GlcNSα-4IdoAα-4GlcNSα-4IdoAα-<br>SU-2 6-SU SU-2 SU-6 SU-2 SU-6 SU-2 SU-6 SU-2 SU-6 SU-2<br>-4GlcNSα-4IdoAα-4GlcNS-AO*<br>SU-6 SU-2 SU-6	-	-
376	HS-S4-AO	GlcAß-4GlcNAcα-4GlcAß-4aMan-AO*<br>(Variously 6S and NS?)	-	-
377	HS-S8-AO	GlcAß-4GlcNAcα-4GlcAß-4GlcNAcα-4GlcAGlcAß-4GlcNAcα-4GlcAß-4aMan-AO*<br>(Variously 6S and NS?)	-	-
378	GN2	GlcNAcß-4GlcNAc	-	-
379	GN2.AO	GlcNAcß-4GlcNAc-AO	-	-
380	GN3	GlcNAcß-4GlcNAcß-4GlcNAc	-	-
381	GN3.AO	GlcNAcß-4GlcNAcß-4GlcNAc-AO	-	-
382	GN4.AO	GlcNAcß-4GlcNAcß-4GlcNAcß-4GlcNAc-AO	-	-
383	GN5.AO	GlcNAcß-4GlcNAcß-4GlcNAcß-4GlcNAcß-4GlcNAc-AO	-	-
384	GN6.AO	GlcNAcß-4GlcNAcß-4GlcNAcß-4GlcNAcß-4GlcNAcß-4GlcNAc-AO	-	-
385	GN7.AO	GlcNAcß-4GlcNAcß-4GlcNAcß-4GlcNAcß-4GlcNAcß-4GlcNAcß-4GlcNAc-AO*	-	-
386	GN8.AO	GlcNAcß-4GlcNAcß-4GlcNAcß-4GlcNAcß-4GlcNAcß-4GlcNAcß-4GlcNAcß-4GlcNAc-AO*	-	-
387	Man4(ß4)	Manß-4Manß-4Manß-4Man	-	-
388	Man5(ß4)	Manß-4Manß-4Manß-4Manß-4Man	-	-
389	Man6(ß4)	Manß-4Manß-4Manß-4Manß-4Manß-4Man	-	-
390	Xyl5(ß4)	Xylß-4Xylß-4Xylß-4Xylß-4Xyl	-	-
391	Xyl6(ß4)	Xylß-4Xylß-4Xylß-4Xylß-4Xylß-4Xyl	-	-
392	Ara6(α5)	Araα-5Araα-5Araα-5Araα-5Araα-5Ara	-	-
393	Ara7(α5)	Araα-5Araα-5Araα-5Araα-5Araα-5Araα-5Ara	-	-
394	Glc2(α2)-AO	Glcα-2Glc-AO	-	-
395	Glc2(α3)-AO	Glcα-3Glc-AO	-	-

396	Malto-2-AO	Glcα-4Glc-AO	-	-
397	Malto-3-AO	Glcα-4Glcα-4Glc-AO	-	-
398	Malto-4-AO	Glcα-4Glcα-4Glcα-4Glc-AO	-	-
399	Malto-5-AO	Glcα-4Glcα-4Glcα-4Glcα-4Glc-AO	-	-
400	Malto-6-AO	Glcα-4Glcα-4Glcα-4Glcα-4Glcα-4Glc-AO	-	-
401	Malto-7-AO	Glcα-4Glcα-4Glcα-4Glcα-4Glcα-4Glcα-4Glc-AO	-	-
402	Malto-8-AO	Glcα-4Glcα-4Glcα-4Glcα-4Glcα-4Glcα-4Glcα-4Glc-AO*	-	-
403	Malto-9-AO	Glcα-4Glcα-4Glcα-4Glcα-4Glcα-4Glcα-4Glcα-4Glcα-4Glc-AO*	-	-
404	Malto-10-AO	Glcα-4Glcα-4Glcα-4Glcα-4Glcα-4Glcα-4Glcα-4Glcα-4Glcα-4Glc-AO*	-	-
405	Malto-11-AO	Glcα-4Glcα-4Glcα-4Glcα-4Glcα-4Glcα-4Glcα-4Glcα-4Glcα-4Glcα-4Glc-AO*	-	-
406	Malto-12-AO	Glcα-4Glcα-4Glcα-4Glcα-4Glcα-4Glcα-4Glcα-4Glcα-4Glcα-4Glcα-4Glcα-4Glc-AO*	-	-
407	Malto-13-AO	Glcα-4Glcα-4Glcα-4Glcα-4Glcα-4Glcα-4Glcα-4Glcα-4Glcα-4Glcα-4Glcα-4Glcα-4Glc-AO*	-	-
408	Dextran-2-AO	Glcα-6Glc-AO	-	-
409	Dextran-3-AO	Glcα-6Glcα-6Glc-AO	-	-
410	Dextran-4-AO	Glcα-6Glcα-6Glcα-6Glc-AO	-	-
411	Dextran-5-AO	Glcα-6Glcα-6Glcα-6Glcα-6Glc-AO	-	-
412	Dextran-6-AO	Glcα-6Glcα-6Glcα-6Glcα-6Glcα-6Glc-AO	-	8
413	Dextran-7-AO	Glcα-6Glcα-6Glcα-6Glcα-6Glcα-6Glcα-6Glc-AO	-	59
414	Dextran-8-AO	Glcα-6Glcα-6Glcα-6Glcα-6Glcα-6Glcα-6Glcα-6Glc-AO*	-	166
415	Dextran-9-AO	Glcα-6Glcα-6Glcα-6Glcα-6Glcα-6Glcα-6Glcα-6Glcα-6Glc-AO*	-	74
416	Dextran-10-AO	Glcα-6Glcα-6Glcα-6Glcα-6Glcα-6Glcα-6Glcα-6Glcα-6Glcα-6Glc-AO*	-	60
417	Dextran-11-AO	Glcα-6Glcα-6Glcα-6Glcα-6Glcα-6Glcα-6Glcα-6Glcα-6Glcα-6Glcα-6Glc-AO*	-	145
418	Dextran-12-AO	Glcα-6Glcα-6Glcα-6Glcα-6Glcα-6Glcα-6Glcα-6Glcα-6Glcα-6Glcα-6Glcα-6Glc-AO*	-	109
419	Dextran-13-AO	Glcα-6Glcα-6Glcα-6Glcα-6Glcα-6Glcα-6Glcα-6Glcα-6Glcα-6Glcα-6Glcα-6Glcα-6Glc-AO*	-	37
420	Lam-2-AO	Glcß-3Glc-AO	-	-
421	Lam-3-AO	Glcß-3Glcß-3Glc-AO	-	-
422	Lam-4-AO	Glcß-3Glcß-3Glcß-3Glc-AO	-	-
423	Lam-5-AO	Glcß-3Glcß-3Glcß-3Glcß-3Glc-AO	-	-
424	Lam-6-AO	Glcß-3Glcß-3Glcß-3Glcß-3Glcß-3Glc-AO	-	-

425	Lam-7-AO	Glcß-3Glcß-3Glcß-3Glcß-3Glcß-3Glcß-3Glc-AO	-	-
426	Curd-8-AO	Glcß-3Glcß-3Glcß-3Glcß-3Glcß-3Glcß-3Glcß-3Glc-AO*	-	-
427	Curd-9-AO	Glcß-3Glcß-3Glcß-3Glcß-3Glcß-3Glcß-3Glcß-3Glcß-3Glc-AO*	-	-
428	Curd-10-AO	Glcß-3Glcß-3Glcß-3Glcß-3Glcß-3Glcß-3Glcß-3Glcß-3Glcß-3Glc-AO*	-	-
429	Curd-11-AO	Glcβ-3Glcβ-3Glcβ-3Glcβ-3Glcβ-3Glcβ-3Glcβ-3Glcβ-3Glcβ-3Glcβ-3Glc-AO*	-	-
430	Curd-12-AO	Glcß-3Glcß-3Glcß-3Glcß-3Glcß-3Glcß-3Glcß-3Glcß-3Glcß-3Glcß-3Glcß-3Glc-AO*	-	-
431	Curd-13-AO	Glcβ-3Glcβ-3Glcβ-3Glcβ-3Glcβ-3Glcβ-3Glcβ-3Glcβ-3Glcβ-3Glcβ-3Glcβ-3Glcβ-3Glc-AO*	-	-
432	Cello-2-AO	Glcß-4Glc-AO	-	-
433	Cello-3-AO	Glcß-4Glcß-4Glc-AO	-	-
434	Cello-4-AO	Glcß-4Glcß-4Glcß-4Glc-AO	-	-
435	Cello-5-AO	Glcß-4Glcß-4Glcß-4Glcß-4Glc-AO	-	-
436	Cello-6-AO	Glcß-4Glcß-4Glcß-4Glcß-4Glcß-4Glc-AO	-	-
437	Cello-7-AO	Glcß-4Glcß-4Glcß-4Glcß-4Glcß-4Glcß-4Glc-AO	-	-
438	Cello-8-AO	Glcß-4Glcß-4Glcß-4Glcß-4Glcß-4Glcß-4Glcß-4Glc-AO*	-	-
439	Cello-9-AO	Glcß-4Glcß-4Glcß-4Glcß-4Glcß-4Glcß-4Glcß-4Glcß-4Glc-AO*	-	-
440	Cello-10-AO	Glcß-4Glcß-4Glcß-4Glcß-4Glcß-4Glcß-4Glcß-4Glcß-4Glcß-4Glc-AO*	-	-
441	Cello-11-AO	Glcß-4Glcß-4Glcß-4Glcß-4Glcß-4Glcß-4Glcß-4Glcß-4Glcß-4Glcß-4Glc-AO*	-	-
442	Cello-12-AO	Glcß-4Glcß-4Glcß-4Glcß-4Glcß-4Glcß-4Glcß-4Glcß-4Glcß-4Glcß-4Glcß-4Glc-AO*	-	-
443	Cello-13-AO	Glcß-4Glcß-4Glcß-4Glcß-4Glcß-4Glcß-4Glcß-4Glcß-4Glcß-4Glcß-4Glcß-4Glcß-4Glc-AO*	-	-
444	Pust-2-AO	Glcß-6Glc-AO	-	-
445	Pust-3-AO	Glcß-6Glcß-6Glc-AO	-	-
446	Pust-4-AO	Glcß-6Glcß-6Glcß-6Glc-AO	-	-
447	Pust-5-AO	Glcß-6Glcß-6Glcß-6Glcß-6Glc-AO	-	-
448	Pust-6-AO	Glcß-6Glcß-6Glcß-6Glcß-6Glcß-6Glc-AO	-	-
449	Pust-7-AO	Glcß-6Glcß-6Glcß-6Glcß-6Glcß-6Glcß-6Glc-AO	-	-
450	Pust-8-AO	Glcß-6Glcß-6Glcß-6Glcß-6Glcß-6Glcß-6Glcß-6Glc-AO*	-	-
451	Glc2ß(2).AO	Glcß-2Glc-AO	-	-
Miscellaneous				
452	Gal	Gal	-	-
453	Gal-AO	Gal-AO	-	-
454	GalNAc	GalNAc	-	-
455	GalNAc-AO	GalNAc-AO	-	-
456	Glc	Glc	-	-
457	Glc-AO	Glc-AO	-	-
458	GN	GlcNAc	-	-

459	GN-AO	GlcNAc-AO	-	-
460	Man	Man	-	-
461	Man-AO	Man-AO	-	-
462	Fuc	Fuc	-	-
463	Fuc-AO	Fuc-AO	-	-
464	NeuAc	NeuAc	-	-
465	NeuAc-AO	NeuAc-AO	-	-
466	NeuGc	NeuGc	-	-
467	NeuGc-AO	NeuGc-AO	-	-
468	Rha	Rha	-	-
469	Rha-AO	Rha-AO	-	-
470	Galα-6Glc-AO	Galα-6Glc-AO	-	-
471	(6P)-Glc-AO	P-6Glc-AO	-	-
472	(6P)-Man	P-6Man	-	-
473	(6P)-Man-AO	P-6Man-AO	-	-
474	(6P)-Man5	P-6Manα-3Manα-3Manα-3Manα-2Man	-	-
475	(6P)-Fructose-AO	P-6Fructose-AO	-	-
476	SU-Tyr	SU-Tyr	-	-
477	SU-Cholesterol	SU-Cholesterol	-	-
478	GN-Asn	GlcNAc-Asn	-	-
479	GlcNAcß1-2Fuc-AO	GlcNAcß1-2Fuc-AO	-	-
480	GlcNAcß1-4Fuc-AO	GlcNAcß1-4Fuc-AO	-	-
481	Xyl3Glc4	Glcß-4Glcß-4Glcß-4Glc<br>| | | Xylα-6 Xylα-6 Xylα-6	-	-
482	GSC-284	GalNAcß-6Galß-4Glcß-Cer36<br>| NeuAcα-3	-	-
483	GSC-575	GalNAcß-4Galß-3Galß-C30<br>| NeuAcα-3	-	-
484	GSC-70	NeuAcα-6Galß-6GalNAcß-4Galß-4Glcß-Cer36	-	-
485	GSC-154	NeuAcα-3Galß-4GlcNAcß-6Galß-4Glcß-Cer36<br>| Fucα-3	-	-
486	GSC-446	NeuAcα-3Galß-4GlcNAcß-6GalNAcα-3Galß-4Glc-C30	-	-
487	GSC-441	NeuAcα-3Galß-4GlcNAcß-6GalNAcα-3Galß-4Glcß-C30	-	-

488	GSC-384	NeuAcα-3Galß-4GlcNAcß-4GalNAcß-3Galß-4Glcß-C30 | Fucα-3	8	-
489	Glc(α6,α4,α4)	Glcα-6Glcα-4Glcα-4Glc	-	-
490	Glc(α6,α4,α4)-AO	Glcα-6Glcα-4Glcα-4Glc-AO	-	-
491	O1.AO	GlcNAcß-3 | Gal-AO | GlcNAcß-6	-	-
492	Rutinose.AO	Rhaα-gGlc-AO	-	-

[a]Pos, Probe position in the screening microarray.

[b]The oligosaccharide probes are all lipid-linked, neoglycolipids (NGLs) or glycosylceramides and are from the collection assembled in the course of research in Glycosciences Laboratory. Unless otherwise specified the NGLs are prepared from reducing oligosaccharides by reductive amination with the amino lipid, 1,2-dihexadecyl-*sn*-glycero-3-phosphoethanolamine (DHPE) (Chai,W, et al, Methods Enzymol. 362, 160-195, 2003); AO, NGLs prepared from reducing oligosaccharides by oxime ligation with an aminooxy (AO) functionalized DHPE (Liu et al., Chem. Biol. 14, 847–859, 2007); Cer, natural glycolipids with various ceramide moieties; CerA and CerB denote different natural ceramides; Cer36 and Cer42, synthetic glycolipids with ceramide having a total of 32 and 42 carbon atoms, respectively; C30, a synthetic lipid [2-(tetradecyl)hexadecanol] with 30 carbon atoms. UA, 4,5-unsaturated hexuronic acid; aMan, 2,5-anhydro-mannose; aGal, 3,6-anhydro-galactose.

[c]Less than 1.

*Major component.

Supplemental Table S2 **Compositions of the reductively released *O*-glycans in the Bio-Gel P6 fractions 1-7, deduced from MALDI-MS analysis.**

Fractions	$[M+Na]^+$	Composition
7		
	814	Hex1.HexNAc2.HexNAcol
	919	Fuc1.Hex2.HexNAc1.HexNAcol
	935	Hex3.HexNAc1.HexNAcol
	960	Fuc1.Hex1.HexNAc2.HexNAcol
	976	Hex2.HexNAc2.HexNAcol
	1017	Hex1.HexNAc3.HexNAcol
	1065	Fuc2.Hex2.HexNAc1.HexNAcol
	1081	Fuc1.Hex3.HexNAc1.HexNAcol
	1122	Fuc1.Hex2.HexNAc2.HexNAcol
	1138	Hex3.HexNAc2.HexNAcol
	1179	Hex2.HexNAc3.HexNAcol
6		
	1122	Fuc1.Hex2.HexNAc2.HexNAcol
	1179	Hex2.HexNAc3.HexNAcol
	1325	Fuc1.Hex2.HexNAc3.HexNAcol
5		
	1122	Fuc1.Hex2.HexNAc2.HexNAcol
	1179	Hex2.HexNAc3.HexNAcol
	1284	Fuc1.Hex3.HexNAc2.HexNAcol
	1325	Fuc1.Hex2.HexNAc3.HexNAcol
	1430	Fuc2.Hex3.HexNAc2.HexNAcol
4		
	1179	Hex2.HexNAc3.HexNAcol
	1284	Fuc1.Hex3.HexNAc2.HexNAcol
	1325	Fuc1.Hex2.HexNAc3.HexNAcol
	1341	Hex3.HexNAc3.HexNAcol
	1382	Hex2.HexNAc4.HexNAcol
	1430	Fuc2.Hex3.HexNAc2.HexNAcol
	1471	Fuc2.Hex2.HexNAc3.HexNAcol
	1487	Fuc1.Hex3.HexNAc3.HexNAcol
	1503	Hex4.HexNAc3.HexNAcol
	1633	Fuc2.Hex3.HexNAc3.HexNAcol
	1690	Fuc1.Hex3.HexNAc4.HexNAcol
3		
	1325	Fuc1.Hex2.HexNAc3.HexNAcol
	1382	Hex2.HexNAc4.HexNAcol
	1430	Fuc2.Hex3.HexNAc2.HexNAcol
	1471	Fuc2.Hex2.HexNAc3.HexNAcol
	1487	Fuc1.Hex3.HexNAc3.HexNAcol
	1544	Hex3.HexNAc4.HexNAcol
	1633	Fuc2.Hex3.HexNAc3.HexNAcol
	1649	Fuc1.Hex4.HexNAc3.HexNAcol
	1690	Fuc1.Hex3.HexNAc4.HexNAcol
	1795	Fuc2.Hex4.HexNAc3.HexNAcol
2		
	1487	Fuc1.Hex3.HexNAc3.HexNAcol
	1544	Hex3.HexNAc4.HexNAcol
	1633	Fuc2.Hex3.HexNAc3.HexNAcol
	1690	Fuc1.Hex3.HexNAc4.HexNAcol
	1747	Hex3.HexNAc5.HexNAcol
	1795	Fuc2.Hex4.HexNAc3.HexNAcol
	1894	Fuc1.Hex3.HexNAc5.HexNAcol
	1852	Fuc1.Hex4.HexNAc4.HexNAcol
	1942	Fuc3.Hex4.HexNAc3.HexNAcol
	1999	Fuc2.Hex4.HexNAc4.HexNAcol
	2055	Fuc1.Hex4.HexNAc5.HexNAcol
1		
	1747	Hex3.HexNAc5.HexNAcol
	1836	Fuc2.Hex3.HexNAc4.HexNAcol
	1852	Fuc1.Hex4.HexNAc4.HexNAcol
	1894	Fuc1.Hex3.HexNAc5.HexNAcol
	1909	Hex4.HexNAc5.HexNAcol
	1998	Fuc2.Hex4.HexNAc4.HexNAcol
	2055	Fuc1.Hex4.HexNAc5.HexNAcol
	2112	Hex4.HexNAc6.HexNAcol
	2160	Fuc2.Hex5.HexNAc4.HexNAcol
	2201	Fuc2.Hex4.HexNAc5.HexNAcol
	2258	Fuc1.Hex4.HexNAc6.HexNAcol
	2306	Fuc3.Hex5.HexNAc4.HexNAcol
	2364	Fuc2.Hex5.HexNAc5.HexNAcol
	2405	Fuc2.Hex4.HexNAc6.HexNAcol
	2421	Fuc1.Hex5.HexNAc6.HexNAcol
	2510	Fuc3.Hex5.HexNAc5.HexNAcol
	2567	Fuc2.Hex5.HexNAc6.HexNAcol

Abbreviations: dHex, deoxyhexose; Hex, hexose; HexNAc, *N*-acetylhexosamine, HexNAcol, *N*-acetylhexosaminitol.

Supplemental Table S3 **Compositions of the NGLs derived from the reductively released *O*-glycans in Bio-Gel P6 fractions 1-7, deduced from MALDI-MS analysis.**

Fractions	[M+H]+	Composition
7		
	1218	Hex1-OX
	1364	Fuc1.Hex1-OX
	1421	Hex1.HexNAc1-OX
	1466	Fuc1.Hex1.HexNAc1-OY
	1523	Hex1.HexNAc2-OY
6		
	1219	Hex1-OX
	1365	Fuc1.Hex1-OX
	1422	Hex1.HexNAc1-OX
	1467	Fuc1.Hex1.HexNAc1-OY
	1524	Hex1.HexNAc2-OY
5		
	955	-OY
	1158	HexNAc1-OY
	1422	Hex1,HexNAc1-OX
	1524	Hex1.HexNAc2-OY
	1568	Fuc1.Hex1.HexNAc1-OX
	1670	Fuc1.Hex1.HexNAc2-OY
	1787	Hex2.HexNAc2-OX
	1934	Fuc1.Hex2.HexNAc2-OX
4		
	955	-OY
	1158	HexNAc1-OY
	1321	Hex1.HexNAc1-OY
	1422	Hex1,HexNAc1-OX
	1524	Hex1.HexNAc2-OY
	1568	Fuc1.Hex1.HexNAc1-OX
	1670	Fuc1.Hex1.HexNAc2-OY
	1730	Fuc1.Hex2.HexNAc1-OX
	1787	Hex2.HexNAc2-OX
	1934	Fuc1.Hex2.HexNAc2-OX
	1991	Hex2.HexNAc3-OX
	2095	Fuc1.Hex3.HexNAc2-OX
	2241	Fuc2.Hex3.HexNAc2-OX
	2298	Fuc1.Hex3.HexNAc3-OX
	2355	Hex3.HexNAc4-OX
3		
	955	-OY
	1057	-OX
	1158	HexNAc1-OY
	1321	Hex1.HexNAc1-OY
	1364	Fuc1.Hex1-OX
	1422	Hex1,HexNAc1-OX
	1467	Fuc1.Hex1.HexNAc1-OY
	1524	Hex1.HexNAc2-OY
	1568	Fuc1.Hex1.HexNAc1-OX
	1670	Fuc1.Hex1.HexNAc2-OY
	1730	Fuc1.Hex2.HexNAc1-OX
	1787	Hex2.HexNAc2-OX
	1890	Hex2.HexNAc3-OY
	1934	Fuc1.Hex2.HexNAc2-OX
	1991	Hex2.HexNAc3-OX
	2095	Fuc1.Hex3.HexNAc2-OX
	2241	Fuc2.Hex3.HexNAc2-OX
	2298	Fuc1.Hex3.HexNAc3-OX
2		
	955	-OY
	1057	-OX
	1158	HexNAc1-OY
	1218	Hex1-OX
	1321	Hex1.HexNAc1-OY
	1364	Fuc1.Hex1-OX
	1422	Hex1.HexNAc1-OX
	1467	Fuc1.Hex1.HexNAc1-OY
	1524	Hex1.HexNAc2-OY
	1670	Fuc1.Hex1.HexNAc2-OY
	1730	Fuc1.Hex2.HexNAc1-OX
	1787	Hex2.HexNAc2-OX
	1890	Hex2.HexNAc3-OY
	1934	Fuc1.Hex2.HexNAc2-OX
	1991	Hex2.HexNAc3-OX
	2035	Fuc1.Hex2.HexNAc3-OY
	2095	Fuc1.Hex3.HexNAc2-OX
	2137	Fuc1.Hex2.HexNAc3-OX
	2153	Hex3.HexNAc3-OX
	2241	Fuc2.Hex3.HexNAc2-OX
	2298	Fuc1.Hex3.HexNAc3-OX
	2355	Hex3.HexNAc4-OX
	2445	Fuc2.Hex3.HexNAc3-OX
	2505	Fuc1.Hex3.HexNAc4-OX
	2648	Fuc2.Hex3.HexNAc4-OX
	2693	Fuc2.Hex2.HexNAc5-OX
1		
	955	-OY
	1158	HexNAc1-OY
	1218	Hex1-OX
	1321	Hex1.HexNAc1-OY
	1422	Hex1 ,HexNAc1-OX
	1467	Fuc1.Hex1.HexNAc1-OY
	1524	Hex1.HexNAc2-OY
	1568	Fuc1.Hex1.HexNAc1-OX
	1670	Fuc1.Hex1.HexNAc2-OY
	1787	Hex2.HexNAc2-OX
	1934	Fuc1 ,Hex2.HexNAc2-OX
	1991	Hex2.HexNAc3-OX
	2137	Fuc1 ,Hex2.HexNAc3-OX
	2298	Fuc1 ,Hex3.HexNAc3-OX
	2355	Hex3.HexNAc4-OX
	2399	Hex2.HexNAc5-OX
	2445	Fuc2.Hex3.HexNAc3-OX
	2501	Fuc1.Hex3.HexNAc4-OX
	2545	Fuc1.Hex2.HexNAc5-OX
	2607	Fuc2.Hex4.HexNAc3-OX
	2648	Fuc2.Hex3.HexNAc4-OX
	2692	Fuc2.Hex2.HexNAc5-OX
	2749	Fuc1.Hex2.HexNAc6-OX
	2811	Fuc2.Hex4.HexNAc4-OX

Abbreviations: dHex, deoxyhexose; Hex, hexose; HexNAc, *N*-acetylhexosamine, HexNAcol, *N*-acetylhexosaminitol. –OX and –OY are the 3- and 6- linked fragments of core GalNAc after periodate oxidation as further explained in footnote of Table 3**.**

Supplemental Table S4 **Oligosaccharide probes included in the newly generated F77/Ii focused array and the binding signals (means of the fluorescence intensities) elicited with mAb F77 at 5 fmol probe per spot (taken from Fig 7).**

Pos[a]	Probe[b]	Sequence	Antigen activity	Binding Signals of mAb F77 (5fmol/probe spot)
1	LNFP-I	Fucα-2Galß-3GlcNAcß-3Galß-4Glc-DH	Linear H type 1	-[c]
2	LnNFPI	Fucα-2Galß-4GlcNAcß-3Galß-4Glc-DH	Linear H type 2	-
3	H2 (& H2+Fuc)	Fucα-2Galß-4GlcNAcß-3Galß-4GlcNAcß-3Galß-4GlcNAcß-Cer	Linear H type 2	-
4	H3 (& H3-Fuc)	Galß-4GlcNAcß-6<br>Fucα-2 Galß-4GlcNAcß-3Galß-4Glc-Cer<br>Galß-4GlcNAcß-3<br>Fucα-2	Branched H type 2	34711
5	A-Hexa-T1	GalNAcα-3Galß-3GlcNAcß-3Galß-4Glc-DH<br>Fucα-2	Linear A type 1	-
6	A-Hexa-T2	GalNAcα-3Galß-4GlcNAcß-3Galß-4Glc-DH<br>Fucα-2	Linear A type 2	-
7	Ab (& Ab+Fuc)	GalNAcα-3Galß-4GlcNAcß-3Galß-4GlcNAcß-3Galß-4Glcß-Cer<br>Fucα-2	Linear A type 2	-
8	Ad (& Ad+Fuc)	GalNAcα-3Galß-4GlcNAcß-6<br>Fucα-2 Galß-4GlcNAcß-3Galß-4Glcß-Cer<br>GalNAcα-3Galß-4GlcNAcß-3Galß-4GlcNAcß-3<br>Fucα-2	Branched A type 2	24972
9	B-Hexa-T1	Galα-3Galß-3GlcNAcß-3Galß-4Glc-DH<br>Fucα-2	Linear B type 1	-
10	B-Hexa-T2	Galα-3Galß-4GlcNAcß-3Galß-4Glc-DH<br>Fucα-2	Linear B type 2	-

<table>
<tr><td>11</td><td>B-III dodeca</td><td>Galα-3Galß-4GlcNAcß-6
| |
Fucα-2 Galß-4GlcNAcß-3Galß-4Glcß-Cer
|
Galα-3Galß-4GlcNAcß-3
|
Fucα-2</td><td>Branched B type 2</td><td>33084</td></tr>
<tr><td>12</td><td>B-IV tetradeca</td><td>Galα-3Galß-4GlcNAcß-6
| |
Fucα-2 Galß-4GlcNAcß-3Galß-4Glcß-Cer
|
Galα-3Galß-4GlcNAcß-3Galß-4GlcNAcß-3
|
Fucα-2</td><td>Branched B type 2</td><td>29777</td></tr>
<tr><td>13</td><td>PSM-4M-f1</td><td>Galß-4GlcNAcß-6
| |
Fucα-2 Galß-4GlcNAcß-OY
|
GlcNAcß-3</td><td>Branched H type 2</td><td>30415</td></tr>
<tr><td>14</td><td>MFLNH-I</td><td>Galß-4GlcNAcß-6
|
Galß-4Glc-DH
|
Galß-3GlcNAcß-3
|
Fucα-2</td><td>Branched H type 1</td><td>6</td></tr>
<tr><td>15</td><td>MSDFLNnH</td><td>Galß-4GlcNAcß-6
| | |
Fucα-2 Fucα-3 Galß-4Glc-AO
|
NeuAcα-6Galß-4GlcNAcß-3</td><td>Branched Le^{y}</td><td>37</td></tr>
<tr><td>16</td><td>TFiLNO(1-2,2,3)</td><td>Galß-3GlcNAcß-3Galß-4GlcNAcß-6
| | |
Fucα-2 Fucα-3 Galß-4Glc-DH
|
Galß-3GlcNAcß-3
|
Fucα-2</td><td>Branched H type 1</td><td>-</td></tr>
</table>

[a]Pos, Probe position in the screening microarray.

[b]The oligosaccharide probes are all lipid-linked, neoglycolipids (NGLs) or glycosylceramides. Unless otherwise specified the NGLs are prepared from reducing oligosaccharides by reductive amination with the amino lipid, 1,2-dihexadecyl-*sn*-glycero-3-phosphoethanolamine (DHPE) (Chai,W, et al, Methods Enzymol. 362, 160-195, 2003); AO, NGL prepared from reducing oligosaccharides by oxime ligation with an aminooxy (AO) functionalized DHPE (Liu et al., Chem. Biol. 14, 847–859, 2007); Cer, natural glycolipids with various ceramide moieties; –OY is the 6-linked fragment of core GalNAc after periodate oxidation as further explained in footnote of Table 3.

[c]Less than 1.
